# Iptacopan Reduces Proteinuria and Stabilizes Kidney Function in C3 Glomerulopathy

**DOI:** 10.1016/j.ekir.2024.10.023

**Published:** 2024-10-28

**Authors:** Carla M. Nester, Ute Eisenberger, Alexandre Karras, Moglie le Quintrec, Liz Lightstone, Manuel Praga, Giuseppe Remuzzi, Maria José Soler, Junhao Liu, Matthias Meier, Ronda Tawfik, Guido Junge, Andrea Biondani, Angelo J. Trapani, Nicholas J.A. Webb, Edwin K.S. Wong

**Affiliations:** 1 Stead Family Children’s Hospital-University of Iowa, Iowa City, Iowa, USA; 2 Department of Nephrology, University Hospital Essen, University of Duisburg-Essen, Essen, Germany; 3 Hôpital Européen Georges Pompidou, Paris, Île-de-France, France; 4 Service de Néphrologie et Transplantation Rénale, Centre Hospitalier Universitaire de Montpellier, Montpellier, France; 5 Department of Immunology and Inflammation, Centre for Inflammatory Disease, Imperial College London, London, UK; 6 Imperial College Renal and Transplant Centre, Imperial College Healthcare NHS Trust, Hammersmith Hospital, London, UK; 7 Department of Medicine, Complutense University, Hospital Universitario 12 de Octubre, Madrid, Spain; 8 Istituto di Ricerche Farmacologiche Mario Negri IRCCS, Bergamo, Italy; 9 Nephrology Department, Hospital Universitari Vall d’Hebron, Barcelona, Catalunya, Spain; 10 Novartis Pharmaceuticals Corporation, East Hanover, New Jersey, USA; 11 Novartis Pharma AG, Basel, Basel-Stadt, Switzerland; 12 Department of Translational Medicine, Novartis Institutes for BioMedical Research, Basel, Basel-Stadt, Switzerland; 13 Newcastle University, Newcastle upon Tyne, UK; 14 National Renal Complement Therapeutics Centre, Royal Victoria Infirmary, Newcastle upon Tyne, UK

**Keywords:** C3 glomerulopathy, C3G, complement pathway, iptacopan, kidney, transplantation

## Abstract

**Introduction:**

C3 glomerulopathy (C3G) is a complex, chronic, ultra rare, progressive primary glomerulonephritis, resulting from alternative complement pathway overactivation, leading to kidney failure in most patients, and frequent recurrence in transplants. Iptacopan (LNP023) is an oral, proximal complement inhibitor specifically targeting factor B, that selectively inhibits the alternative complement pathway.

**Methods:**

This was a phase 2 extension study of 26 adult patients with native kidney (cohort A), or recurrent C3G (post kidney transplantation; cohort B) receiving open label iptacopan.

**Results:**

At 12 months, patients in cohort A had a significant reduction in 24-hour urine protein-to-creatinine ratio (UPCR; 57%; *P* < 0.0001; confidence interval [CI]: 0.31–0.59), an improvement in estimated glomerular filtration rate (eGFR; 6.83 ml/min per 1.73 m^2^; *P* = 0.0174; CI: 1.25–12.40), and an increase in serum C3 levels (geometric mean ratio to baseline: 3.53; *P* < 0.0001; CI: 3.01–4.15). In cohort B, most patients had normal urinary protein excretion at baseline (mean [range] 24-hour UPCR: 121 [9–445]), which was slightly lower by 12 months (21% reduction; CI: 0.48–1.31; *P* = 0.3151). In cohort B at 12 months, mean eGFR was at baseline values (mean change from baseline: −0.96 ml/min per 1.73 m^2^; *P* = 0.7335; CI: −6.60 to 4.69). Cohort B patients had significantly higher serum C3 values at 12 months compared with baseline (ratio:1.96; CI: 1.70–2.27; *P* < 0.0001). In cohorts A + B combined, the median difference in C3 deposit score on renal biopsy from baseline was −7.00 (CI: −12.00 to 4.00;) at 9 to 12 months treatment with iptacopan.

**Conclusion:**

These data provide a clinical rationale for further evaluation of long-term treatment of C3G with iptacopan.

## Graphical abstract

**Figure:**
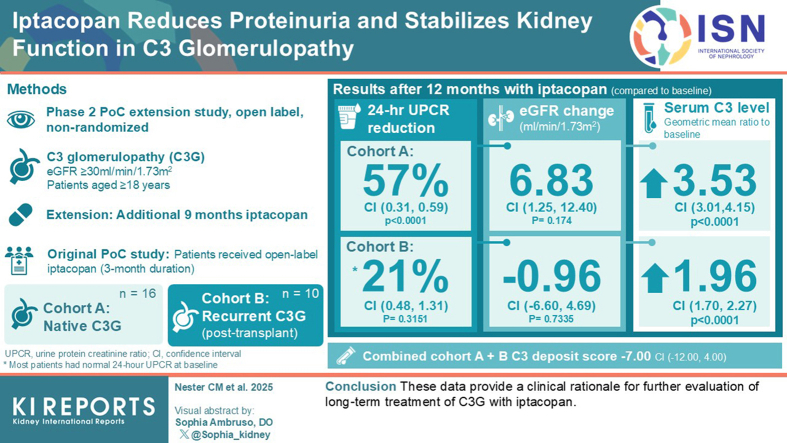

See Commentary on Page 302

C3G is a complex, chronic, progressive, ultra rare form of primary glomerulonephritis, resulting from overactivation of the alternative complement pathway. The estimated worldwide annual incidence approximates 1 to 2 cases per million.^1^^,^^2^ C3G recurs in up to half of all transplanted kidneys.^3^ Overactivation of the alternative pathway (AP) is primarily driven by nephritic factors, autoantibodies that stabilize the C3 and C5 convertases (key alternative complement pathway enzymes), prolonging their half-life and triggering complement hyperactivity.^4^ Uncontrolled AP activation may also be driven by genetic abnormalities in complement genes.^4^, ^5^, ^6^, ^7^ Kidney survival is higher in patients with lower disease chronicity and proteinuria, regardless of baseline eGFR.^8^ Patients with a progressive reduction in proteinuria early during follow-up and over time tend not to reach kidney failure.^9^^,^^10^ A doubling of proteinuria levels results in a 2-fold or more increase in the risk of kidney failure,^10^^,^^11^ whereas reductions of ≥50% in proteinuria within the first 6 or 12 months of follow-up are associated with a decreased risk of kidney failure.^10^ Reductions in proteinuria are also associated with an improvement of eGFR over time,^12^ whereas a faster decline in eGFR was associated with higher probability of kidney failure.^9^ Therefore, reductions in proteinuria may translate into clinical benefits in terms of stable or improved eGFR and higher probability of kidney survival.

Although an optimal treatment strategy for C3G using currently available therapeutics has not been demonstrated or established,^13^ novel therapies targeting the underlying pathogenic mechanisms aim to improve predictors of disease progression (reduction of proteinuria and stabilization or improvement of eGFR) and prevent glomerular and tubulointerstitial inflammation as well as kidney fibrosis in patients with C3G.

Iptacopan is an oral, proximal complement inhibitor that targets factor B selectively, blocking the alternative complement pathway.^14^ This prevents AP-related C3 convertase activity and the subsequent formation of C5 convertase.^15^ By inhibiting the AP, the amplification loop of the entire complement system, iptacopan leaves direct signaling from the lectin and classical pathways intact.^14^

We have previously reported data from a phase 2 proof-of-concept (PoC) study in patients with native and recurrent C3G,^16^ demonstrating the efficacy of iptacopan in targeting the disease-specific pathogenesis in C3G. A 12-week course of iptacopan (200 mg twice daily) resulted in a 45% reduction in proteinuria (*P* = 0.0003) in patients with native kidney C3G, and a reduction in C3 deposit scores (*P* = 0.03) in biopsies of patients with recurrent C3G posttransplantation. Inhibition of AP activity and improvement in eGFR, although nonsignificant, were seen in both C3G cohorts with a favorable safety profile.^16^

Here, we present the findings after 12 months of iptacopan treatment (3 months in the phase 2 PoC study plus 9 months treatment in the extension study) in adults with native kidney (cohort A) as well as recurrent C3G after kidney transplantation (cohort B).

## Methods

### Primary Objectives

The primary efficacy objective in cohort A was to assess the effect of iptacopan on a composite kidney end point of stable or improved eGFR (≤10% reduction from baseline); ≥50% reduction in UPCR from baseline; and ≥50% increase in serum C3 from baseline after 12 months of treatment. The primary efficacy objective in cohort B was to assess the effect of iptacopan treatment on biopsy-proven glomerular C3 deposition after 9 to 12 months of treatment. The primary safety objective was to evaluate the long-term safety and tolerability of iptacopan in all patients with C3G. “Baseline” refers to the day 1 visit (predose) of the phase 2 PoC study.

### Key Secondary and Exploratory Objectives

The key secondary objectives were to assess the long term-effects of iptacopan on kidney function (log-transformed UPCR and eGFR) after 12 months of treatment, changes in serum C3; C3G disease progression based on change in glomerular histopathology (C3 deposit score, total activity, and chronicity scores after 9–12 months of treatment^8^^,^^17^) compared to a pretreatment biopsy; that is those obtained at baseline in the PoC study (if available).

UPCR was measured in g/mol or mg/mmol; 100 mg/mmol equated to proteinuria of 1 g/d.^18^ For subgroup analyses, higher or “nephrotic proportion” baseline proteinuria was defined as UPCR > 339 g/mol, and lower baseline proteinuria was defined as UPCR ≤ 339 g/mol.

A further secondary objective was to evaluate the pharmacokinetics of iptacopan in patients with prolonged treatment by determining iptacopan plasma trough concentrations for up to 12 months of treatment.

Exploratory objectives included evaluating the long-term effect of iptacopan on complement AP activity and complement pathway components (Bb, Wieslab, sC5b-9) and assessing the long-term effect of iptacopan on urine markers of kidney damage lipocalin-2 (neutrophil gelatinase-associated lipocalin [NGAL])/creatinine at the 9-month visit.

### Study Design

**Figure 1:**
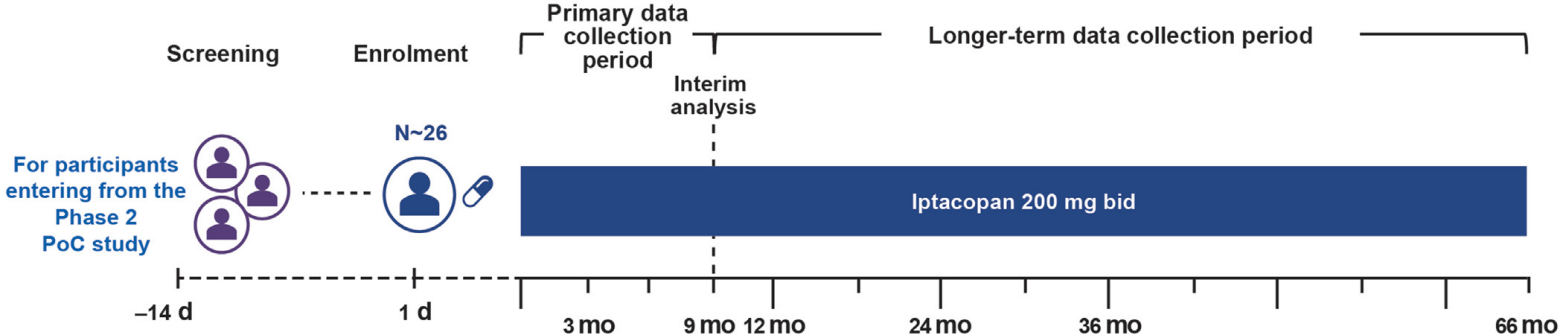
Study design of extension study. 1d refers to the day 1 visit for this extension study. At the final treatment visit of the 3-month phase 2 PoC study, patients who wished to continue to the extension study were asked to review and sign an informed consent form specific to the extension study in advance of the 3-month assessments for the phase 2 PoC study; these data were also used for the day 1 visit of the extension study. At screening for the extension study, inclusion and exclusion criteria were assessed and patients meeting these requirements were enrolled into the extension study and given their first dose of 200 mg bid iptacopan of the extension study. Patients returned to the study center for planned visits approximately every 3 months for the first year, and every 6 months thereafter for the duration of the study. For patients of the phase 2 PoC study, data collection during the open-label treatment period in the extension study was separated into 2 distinct periods, a “primary data collection period” ending at 9 months (i.e., 12 months of continuous treatment including that which occurred in the phase 2 PoC study), and a “longer-term data collection period” beginning at 9 months and continuing until the end of the study. The extension study is anticipated to last up to approximately 66 months. bid, twice daily; d, day; IA, interim analysis; mo, month; PoC, proof of concept.

This was an open-label, nonrandomized, multicenter extension of a C3G phase 2 PoC study (Figure 1); the design has been previously published in more detail.^16^ Briefly, adults with native (cohort A) or recurrent C3G after kidney transplantation (cohort B) received iptacopan for ≥12 weeks in the phase 2 PoC study (NCT03832114) before entering this extension study (NCT03955445). Patients’ consent was sought again before entering the extension study.

### Study Populations

The study populations were defined in the PoC study.^16^ Briefly, all patients (cohorts A and B) had biopsy-confirmed C3G and eGFR ≥ 30 ml/min per 1.73 m^2^; patients in cohort A had proteinuria ≥ 100 mg/mmol despite treatment with angiotensin-converting enzyme inhibitor or angiotensin II receptor blockers and reduced C3 at screening (< 81 mg/dl, i.e., < 0.90 × lower limit of normal laboratory range). Patients with reduced serum C3 levels at screening (< 0.90 × lower limit of normal laboratory range) and with UPCR ≥ 100 g/mol in first morning void urine, or ≥1 g/24 h total urinary protein excretion from a 24-hour urine collection during the run-in period were enrolled in the native cohort. Men and women aged ≥18 years at screening who had completed treatment in the PoC study and who met the inclusion and exclusion criteria for the extension study (Supplementary Methods) were eligible for enrollment in the extension study. Ongoing use of stable doses of mycophenolate acids, such as mycophenolate mofetil or mycophenolate sodium and/or ≤ 7.5 mg/d of prednisolone or equivalent was permitted during the PoC and the extension studies. Exclusion criteria included severe concurrent comorbidities, or any illness or medical condition that in the opinion of the investigator or sponsor was likely to prevent the patients from tolerating iptacopan safely or complying with the requirements of the study; and either an active systemic infection within 14 days before screening, or the presence of fever (≥38 °C or 100.4 °F) within 7 days before screening. The Supplementary Methods include full details of the inclusion and exclusion criteria.

### Histopathology

Biopsies for cohort B were done between 6 and 9 months of the extension study period (i.e., 9–12 months total study period). Baseline biopsies were performed pretreatment and up to 1-year prescreening as described in the previous phase 2 PoC study.^16^ Biopsies were optional for patients in cohort A (native C3G). The glomerular C3 deposit score was based on analysis of kidney biopsy by immunofluorescence microscopy assessed by 3 independent pathologists. C3 deposits were scored separately on a 0 to 3 scale (0, no staining; 1+ mild; 2+ moderate and 3+ strong intensity) for overall average intensity for the mesangial and capillary locations. Each score was multiplied by a factor of 1 for segmental (less than half overall of glomerular capillary walls or mesangial areas positive) and a factor of 2 for global extent (at least half of capillary walls or mesangial areas positive). The total score range was therefore 0 to 12. Disease activity and chronicity were scored as previously described using light microscopy on periodic acid-Schiff-stained slides from light microscopic portions of the biopsies.^8^ The C3 deposit score was used to gauge the effectiveness of iptacopan in preventing glomerular C3 deposition, which is directly linked to C3G pathophysiology and considered causal to kidney impairment. Activity and chronicity scores are indicative of kidney inflammation and fibrosis, respectively.

Given that the number of biopsies was small in both cohorts, analyses were carried out in cohort B alone as well as in cohort A + cohort B.

### Biomarker Assessments

Soluble complement biomarkers C3, Bb (fragment of factor B) and sC5b-9 were measured in plasma or serum using validated assays (nephelometry assay for C3 and enzyme-linked immunosorbent assay for Bb and sC5b-9) as described previously^16^ on day 1 of the extension study and at 3 monthly intervals. Both raw data and changes from baseline at the 9-month visit were analyzed.

### Pharmacokinetics

Trough iptacopan concentrations were measured in plasma using a validated liquid chromatography coupled with tandem mass spectrometry (liquid chromatography-tandem mass spectrometry) method with the lower limit of quantification in plasma of 1 ng/ml.

### Statistical Analysis

This 9-month interim analysis was carried out when the last patient reached the 9-month visit of the extension study, that is, after 12 months of treatment. Data were analyzed using SAS, version 9.4 (SAS Institute Inc., Cary, NC). Analysis sets defined for the statistical analysis were the screened analysis set, including all patients who signed the informed consent for participation in the extension study; the safety analysis set, including all patients who received any study drug and had at least 1 safety assessment in the extension study.

All patients within the safety analysis set were included for the primary efficacy and safety analyses as follows:

1. Cohort A: the number and percentage of patients with C3G meeting the requirements of the 3-point composite kidney end point at 12 months of iptacopan treatment.
2. Cohort B: change from baseline in the C3 deposit score compared with baseline at, or after 9 to 12 months of iptacopan treatment.

The safety analysis set was included for the secondary analyses. A mixed model repeated measures was applied, which allows adjustment for difference in baseline measures.

### Analysis of Primary End Points

1. Cohort A: the statistical evaluation of all primary efficacy data was descriptive. Hypothesis testing was not performed.
2. Cohort B: the Wilcoxon signed rank test was used for C3 deposit score data at the 6- to 9-month visit in the extension study to compare the median difference of change from baseline between periods. The Hodges-Lehmann estimate and 2-sided 95% CI for the median difference were provided.

### Analysis of Secondary End Points

The safety analysis set was included for the secondary analysis.

1. Cohort A: the number of participants meeting the requirements of the composite kidney end point 2 at the day 7, day 14, day 21, day 28, day 36, day 64, and day 84 visits in the phase 2 PoC study and at the 3-month, 6-month, 9 month, and 12-month visits of extension study (with the 9-month visit being of primary interest).
2. Raw and ratio to baseline in first morning void UPCR at the day 7, day 14, day 21, day 36, and day 64 visits in the core study and at the 3-month, 6-month, 9 month, and 12-month visits in the extension study.
3. Raw and ratio to baseline in 24-hour UPCR at the day 28 and day 84 visits in the phase 2 PoC study and at the 9-month visit in the extension study.
4. Raw and change from baseline in eGFR at the day 7, day 14, day 21, day 28, day 36, day 64, and day 84 visits in the phase 2 PoC study and at the 3-month, 6-month, 9 month, and 12-month visits in extension study.
5. Cohort B: change from baseline in the disease activity and chronicity scores (based on light microscopy) compared to baseline in the study at the day 84 visit in the phase 2 PoC study and at the 6- to 9-month visit in extension study.

### Analysis of End Point Exploring the Historical eGFR Data in Cohort A

A generalized linear mixed model, with a common intercept, a pretreatment slope, a change in the slope after iptacopan treatment, and cohort were used to predict the pre-post iptacopan change in eGFR over time. eGFR slope prior to iptacopan treatment and the change in eGFR slope after iptacopan treatment was explored and presented graphically.

## Results

Of 27 patients completing the 12-week phase 2 PoC study,^16^ 26 (*n* = 16 cohort A, *n* = 10 cohort B) entered the extension study and received iptacopan 200 mg twice daily treatment. In Table 1, we show patient demographics and baseline characteristics. In Supplementary Table S1, we show patient disposition.

**Table 1:** Patient demographics and baseline characteristics

Characteristics	Cohort A *n* = 16	Cohort B *n* = 10	Overall *N* = 26
Age (yrs)			
*n*	16	10	26
mean	26.1	35.9	29.8
SD	10.57	18.72	14.73
median	22.0	32.5	24.5
Sex, *n* (%)			
Female	6 (37.5)	2 (20.0)	8 (30.8)
Male	10 (62.5)	8 (80.0)	18 (69.2)
Race, *n* (%)			
White	16 (100.0)	9 (90.0)	25 (96.2)
American Indian or Alaska Native	0 (0.0)	1 (10.0)	1 (3.8)
Ethnicity, *n* (%)			
Hispanic or Latino	0 (0.0)	1 (10.0)	1 (3.8)
Not Hispanic or Latino	16 (100.0)	8 (80.0)	24 (92.3)
Not reported	0 (0.0)	1 (10.0)	1 (3.8)
Weight (kg)			
*n*	16	10	26
mean	65.0	73.1	68.1
SD	9.78	18.28	13.92
Height (cm)			
*n*	16	10	26
mean	174.1	176.3	174.9
SD	5.44	11.46	8.14
Body mass index (kg/m^2^)			
*n*	16	10	26
mean	21.4	23.3	22.1
SD	2.72	3.99	3.32
Disease subtype, *n* (%)			
DDD	2 (12.5)	2 (20.0)	4 (15.4)
C3GN	14 (87.5)	7 (70.0)	21 (80.8)
Missing	0 (0.0)	1 (10.0)	1 (3.8)
Background immunosuppressive therapy (cohort A), *n* (%)			
Yes	7 (43.8)		
Mycophenolate mofetil	6 (37.5)		
Mycophenolic acid	1 (6.3)		
Prednisone	2 (12.5)		
Prednisolone	2 (12.5)		
No	9 (56.3)		
RASi, *n* (%)			
ACEi	12 (75.0)	4 (40.0)	16 (61.5)
ARB	4 (25.0)	2 (20.0)	6 (23.1)
24-h UPCR (g/mol)			
*n*	16	8	24
mean	454.0	121.0	343.0
SD	242.16	188.29	273.41
minimum	199	9	9
median	390.6	18.4	318.7
maximum	1019	445	1019
FMV UPCR (g/mol)			
*n*	16	10	26
mean	408.3	98.0	288.9
SD	304.35	182.55	302.10
minimum	82	5	5
median	323.6	9.0	152.4
maximum	988	493	988
Serum C3 (g/l)			
*n*	16	10	26
mean	0.312	0.588	0.418
SD	0.2224	0.2610	0.2701
minimum	0.02	0.17	0.02
median	0.238	0.550	0.433
maximum	0.69	1.00	1.00
Serum creatinine (μmol/l)			
*n*	16	10	26
mean	134.0	147.4	139.1
SD	60.64	39.30	52.98
minimum	62	94	62
median	115.0	140.4	134.4
maximum	271	205	271
eGFR (ml/min per 1.73 m^2^)			
*n*	16	10	26
mean	70.1	54.0	63.9
SD	35.10	17.17	30.15
minimum	28	27	27
median	64.8	59.6	59.6
maximum	134	74	134

ACEi, angiotensin-converting enzyme inhibitors; ARB, angiotensin 2 receptor blockers; C3GN, complement 3 glomerulonephritis; DDD, dense deposit disease; eGFR, estimated glomerular filtration rate; FMV, first morning void; UPCR, urine protein-creatinine ratio.

The primary end point in cohort A is a 3-point composite kidney end point; for clarity, we present the results for the individual end points first.

### Cohort A

#### UPCR

**Figure 2:**
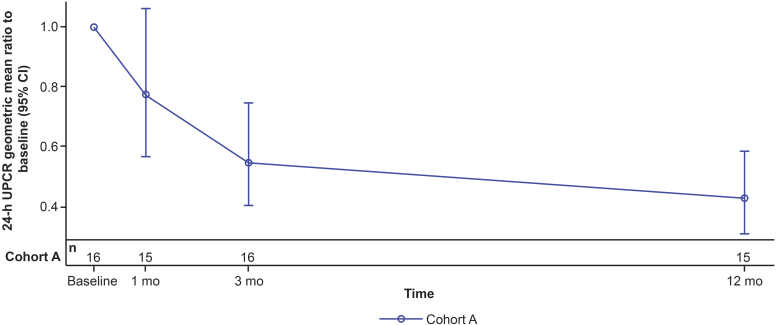
Reduction in 24-hour urine protein-to-creatinine ratio (UPCR) in cohort A. Model-estimated geometric mean ratio to baseline (95% CI) of 24h UPCR (g/mol) from baseline to 12 months.CI, confidence interval; mo, month.

In cohort A, 8 of 15 (53.3%) met the UPCR component of the 3-point composite kidney end point at 12 months. The model-estimated geometric mean ratio to baseline (95% CI) plot of 24-hour UPCR (g/mol) for cohort A is shown in Figure 2. A statistically significant reduction in 24-hour UPCR was observed at 3 months of treatment (45% reduction, *P* = 0.0004, CI: 0.40–0.75) and 12 months (57% reduction; *P* < 0.0001; CI: 0.31–0.59). Overlaying individual time profiles (spaghetti plots) of 24-hour UPCR by cohort are shown in Supplementary Figure S1A. Statistically significant reductions in first morning void UPCR were seen, from 64 days to 12 months (range: 45%–64% decrease) (Supplementary Figure S2).

#### eGFR

**Figure 3:**
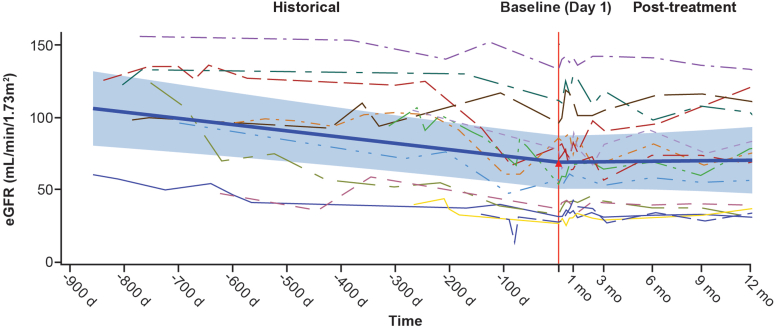
Individual time profiles (spaghetti plots) of eGFR with historical data (cohort A). Only patients with historical data are included. The blue shaded area is 95% confidence band, and the thick blue line is predicted fit. d, day; eGFR, estimated glomerular filtration rate; mo, month.

In cohort A, 93.8% (15/16) of patients met the eGFR component of the 3-point composite kidney end point at 12 months, with a mean change from baseline eGFR of 6.83 ml/min per 1.73 m^2^ (CI: 1.25–12.40; *P* = 0.0174). Individual time profiles (spaghetti plots) of eGFR for patients in cohort A, including historical data from the 2 years before the study are shown in Figure 3; further detail on the results of the analysis of the historical and study data can be found in the Supplementary Results. In cohort A, eGFR increased at all postbaseline visits: mean (SD) eGFR (ml/min per 1.73 m^2^) of 70.09 (35.09) at baseline, 72.67 (36.92) at 3 months, and 76.91 (36.55) at 12 months of iptacopan treatment, reaching statistical significance compared with baseline at 12 months (6.83 ml/min per 1.73 m^2^; *P* = 0.0174; CI: 1.25–12.40) (Supplementary Figure S3).

#### Serum C3

**Figure 4:**
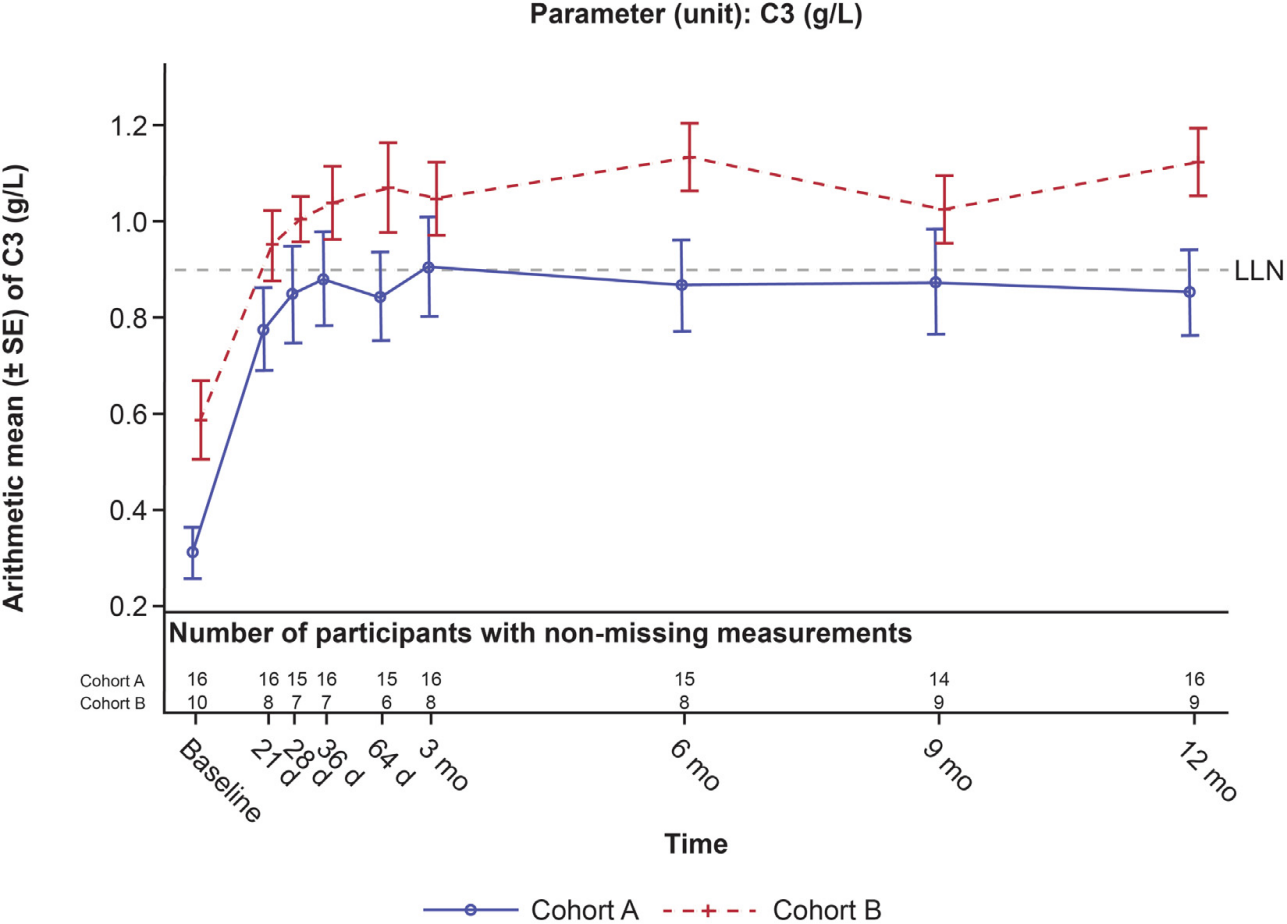
Arithmetic mean (± SE) of serum C3 (g/l) by cohort (safety analysis set). d, day; LLN, lower limit of normal; mo, month; SE, standard error.

All patients (16/16) in cohort A met the C3 criterion of the 3-point composite kidney end point at 12 months. For cohort A, the mean (SD) values of the C3 biomarker (g/l) were 0.31 (0.22) at baseline, 0.91 (0.41) at 3 months, and 0.85 (0.36) at 12 months (Figure 4). At 12 months, the serum C3 level increased to within the normal range in 8 of 16 cohort A participants; individual time profiles of C3 biomarker are shown in Supplementary Figure S4 for cohort A. Statistically significant increases in C3 (model-estimated geometric mean ratio to baseline) were noted in cohort A from 3 to 12 months (range: 3.53–3.61; *P* < 0.0001) at all time points.

#### Composite Kidney End Point

**Figure 5:**
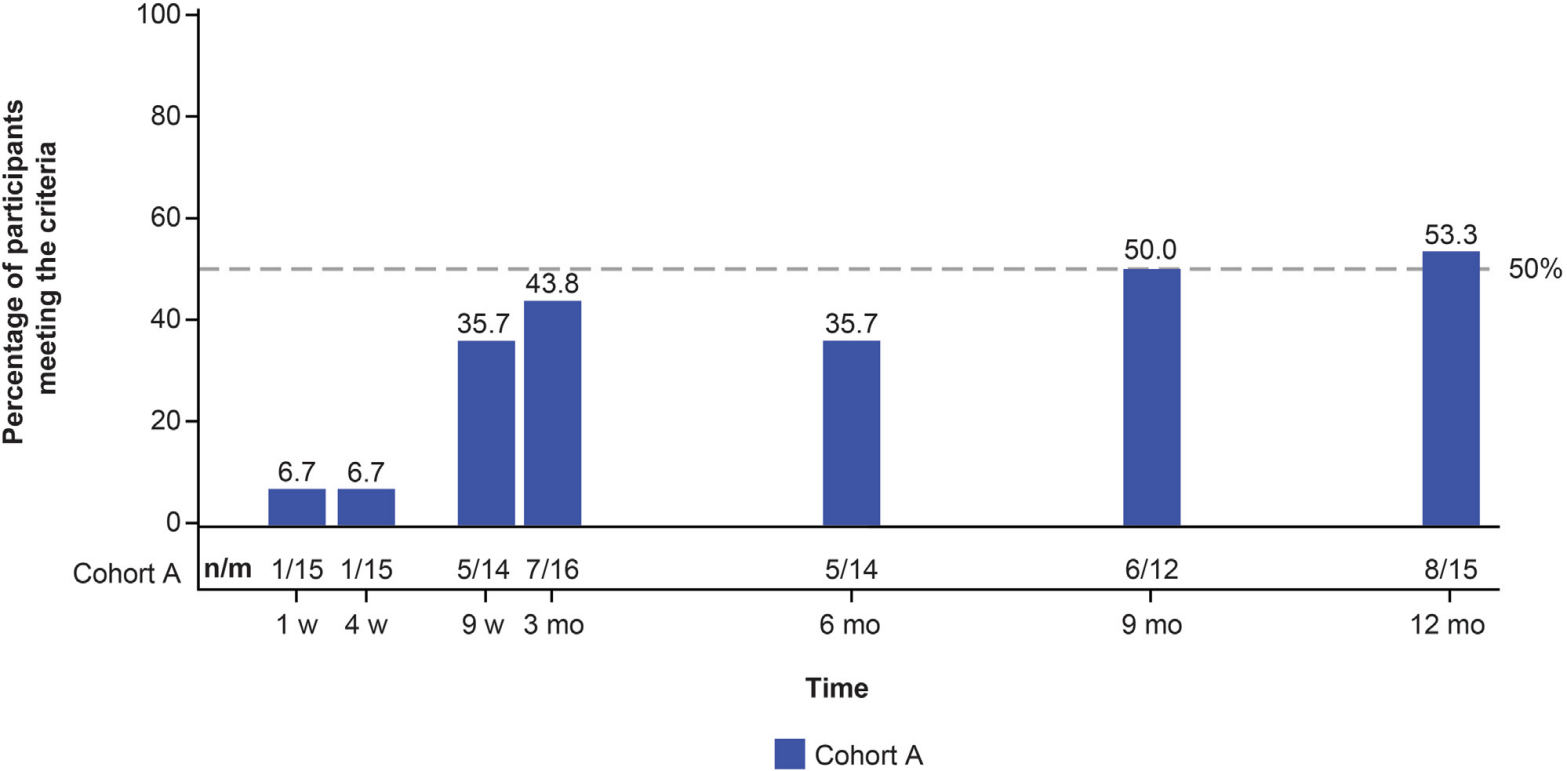
Percentage of patients meeting the criteria of 3-point composite kidney end point over time in cohort A (safety analysis set). A patient was considered to have met the criteria for the 3-point composite kidney end point if they fulfilled all of the individual components: individual component 1: a stable or improved eGFR, defined as ≤10% reduction in eGFR compared to baseline; 2: either a ≥50% reduction in UPCR compared with baseline or a reduction to <300 mg/g in UPCR; 3: either a ≥50% increase in serum C3 compared with baseline or an increase to ≥90 mg/dl in serum C3 (i.e., ≥LLN). bid, twice daily; eGFR, estimated glomerular filtration rate; m, number of patients at each time point; mo, month; *n*, number of patients meeting the criteria at each time point; UPCR, urine protein-to-creatinine ratio; w, week.

After 12 months of treatment with iptacopan 200 mg twice daily, 8 of 15 (53.3%; UPCR data missing for 1 patient at the 12-month visit) of patients in cohort A met the 3-point composite kidney end point criteria. (Figure 5).

#### Subgroup Analyses

Overall, regardless of the level of proteinuria, participants in cohort A had similar reductions in UPCR after 1, 3, and 12 months of iptacopan treatment (Supplementary Table S2).

At 3 months of iptacopan treatment, there was a 47% reduction (CI: 0.35–0.82; *P* = 0.0067) reduction in 24-hour UPCR in patients with “nephrotic proportion” proteinuria and a 43% reduction (CI: 0.34–0.96; *P* = 0.0357) in participants with lower proteinuria. At 12 months, patients with “nephrotic proportion” proteinuria had a significant change in 24-hour UPCR (55% reduction; *P* = 0.0012; CI: 0.29–0.69) as did patients with nonnephrotic proportion proteinuria (62% reduction; *P* = 0.0026; CI: 0.22–0.68).

### Cohort B

#### UPCR

UPCR values were available for 8 of 10 patients in cohort B at baseline: median (range) 24-hour UPCR was 18.4 (9–445) mg/mmol; and mean (SD) 24-hour UPCR was 121.0 (188.29). Of these 8 patients, 6 had proteinuria within the normal range at baseline, which remained low after 1 year of iptacopan treatment. The remaining 2 patients had elevated UPCR at baseline (404.2 mg/mmol and 53.5 mg/mmol), which decreased by 25% and 66%, respectively, at 1 year. Iptacopan treatment was associated with a small, though not statistically significant, reduction in UPCR at 3 months (34% reduction; CI: 0.40–1.08, *P* = 0.0879) and 12 months (21% reduction; CI: 0.48–1.31, *P* = 0.3151). Overlaying individual time profiles (spaghetti plots) of 24-hour UPCR by cohort are shown in Supplementary Figure S1B.

#### eGFR

**Figure 6:**
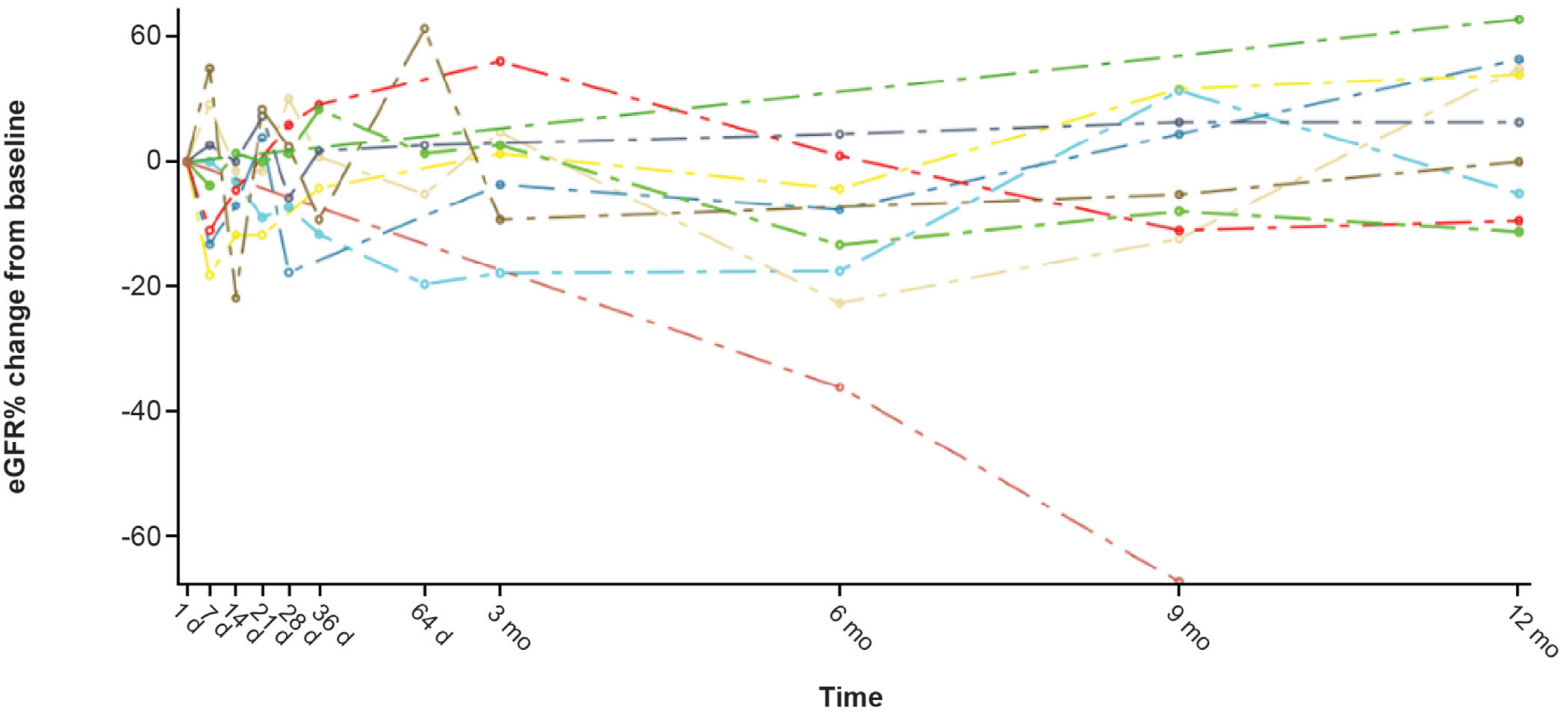
Overlaying individual time profiles of % change from baseline in eGFR (ml/min/1.73 m^2^) in cohort B. d, day; eGFR: estimated glomerular filtration rate; mo, month.

In cohort B, the mean (SD) eGFR values (ml/min per 1.73 m^2^) were 54.78 (14.83) at baseline, 53.76 (17.45) at 3 months, and 53.73 (16.68) at 12 months. One patient in this cohort had a complicated, multimorbid course, including a combination of multiple mental health issues, acute viral infections (cytomegalovirus and COVID-19) and subsequent acute kidney injury. Inclusion of data from this patient accounts for an apparent overall decrease in eGFR at 6 and 9 months. Specifically, a significant decrease in eGFR, compared with baseline, was seen in 6 months (−6.72 ml/min per 1.73 m^2^; *P* = 0.0265; CI: −12.61 to −0.83) and 9 months (−5.64 ml/min per 1.73 m^2^; *P* = 0.0496; CI: −11.27 to −0.01), compared with baseline. In all other patients in cohort B, eGFR stabilized or improved (Figure 6). By 12 months, mean eGFR had returned to baseline values (mean change from baseline: −0.96 ml/min per 1.73 m^2^; *P* = 0.7335; CI: −6.60 to 4.69). Exclusion of the patient above from the analysis results in mean eGFR at 6 and 9 months that is not different from the pretreatment value.

#### Serum C3

For cohort B, the mean (SD) values of C3 biomarker (g/l) were 0.62 (0.29) at baseline, 1.05 (0.21) at 3 months, and 1.12 (0.21) at 12 months. Arithmetic mean (± standard error) of C3 biomarkers by cohort are shown in Figure 4. At 12 months, the C3 level was within the normal range (0.9–1.8 g/l) in 7 of 9 cohort B patients (the 10th patient, described above, withdrew from the study at 9 months because of graft loss). In cohort B, C3 values were significantly higher than baseline from 3 to 12 months of iptacopan treatment (geometric mean ratio range: 1.85–2.09; *P* < 0.0001).

#### Histopathology

**Figure 7:**
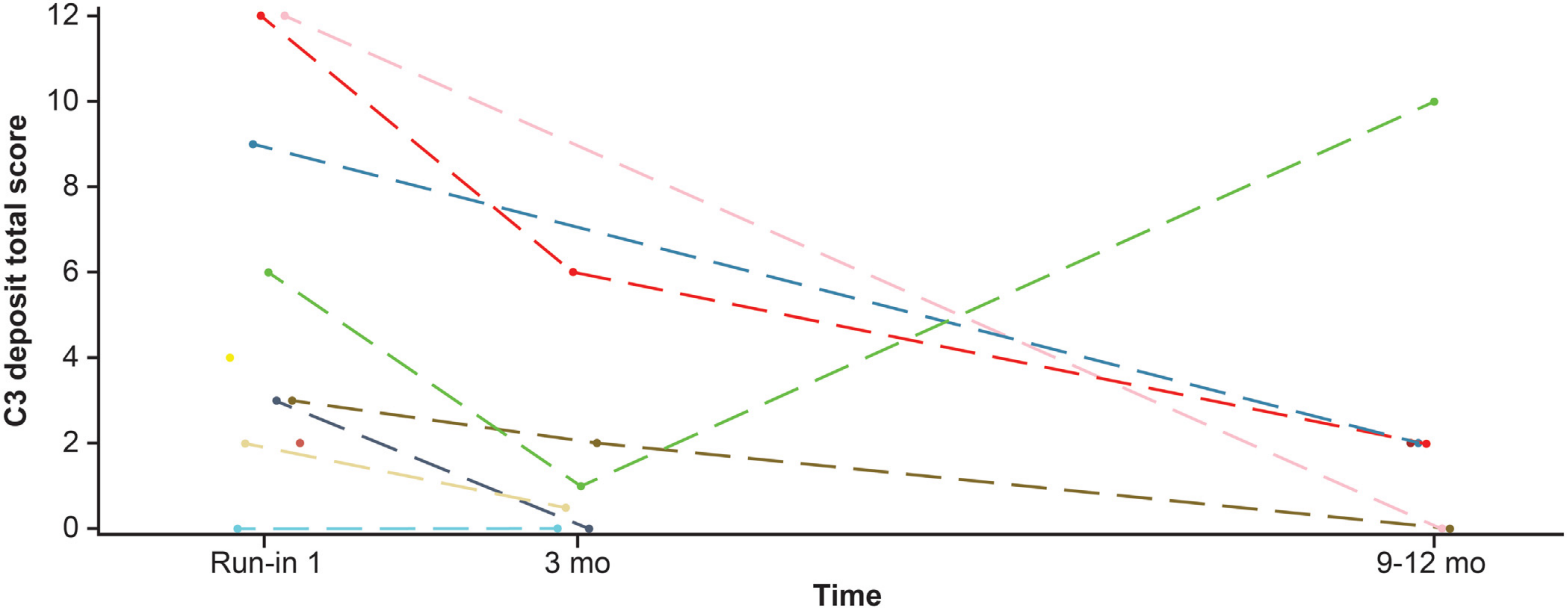
Individual time profiles (spaghetti plots) of C3 deposit total score (cohorts A and B; safety analysis set). C3 deposit intensity was graded on a 0 to 3 scale for the mesangial and capillary location (both scored separately). The score for each location was multiplied by a factor of 1 for segmental (<50%) and a factor of 2 for global (≥50%) extent, resulting in a total score range of 0 to 12. d, day; mo, month.

To assess the effect of iptacopan treatment on glomerular C3 deposition for cohort B, the change from baseline in the C3 deposit score at the 3-month visit in the primary study and at the 6 to 9-month visit in the extension study was compared with baseline for the primary study period. For C3 deposit score in cohort B (recurrent C3G), there were 6 and 4 patients with biopsies at 3 months and 6 to 9 months (i.e., 9–12 months iptacopan treatment), respectively. Examples of the scoring system are shown in Supplementary Figure S5. The median difference in C3 deposit score from baseline (i.e., first biopsy taken) was −2.25 (CI: −6.00 to 0.00; *n* = 6) at 3 months, and −5.50 (CI: −10.00 to 4.00) (*n* = 4) at 6 to 9 months (Supplementary Table S3). Immunofluorescence data from cohort B were limited, because some kidney biopsy samples did not contain glomeruli and were not scored; therefore, an additional analysis was performed incorporating data from patients in both cohorts A (*n* = 1) and B (*n* = 4). In the combined cohort (cohorts A + B), the median difference in C3 deposit score from baseline was −2.25 (CI: −6.00 to 0.00) at 3 months, and −7.00 (CI: −12.00 to 4.00;) at 6 to 9 months (i.e., 9–12 months treatment with iptacopan; Supplementary Table S3). Individual scores are shown in Figure 7.

**Figure 8:**
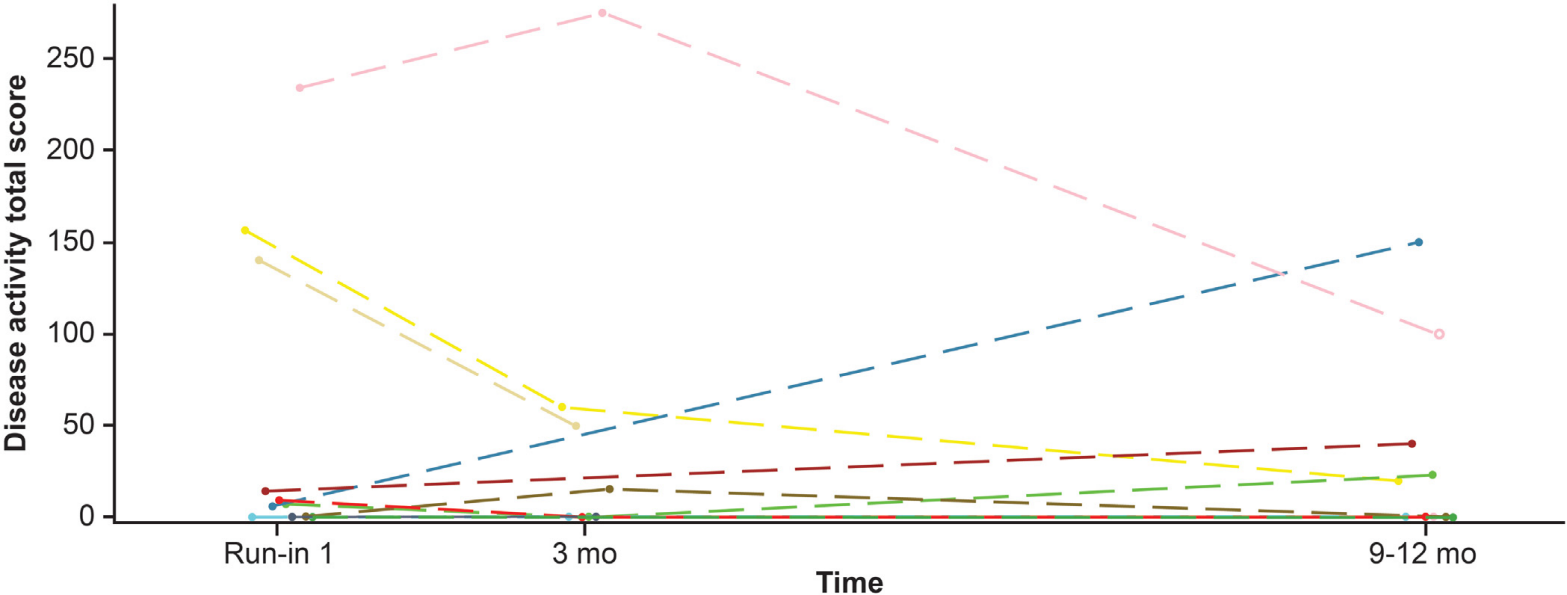
Overlaying individual time profiles (spaghetti plots) of disease activity total score in kidney biopsies (cohorts A and B; safety analysis set). Two patients from cohort A and the remaining profiles are from patients in cohort B. mo, month.

C3G histologic index disease activity and chronicity data from transplant kidney biopsies were available for 7 patients in cohort B and 2 patients in cohort A (biopsy was optional). Most had low activity scores at baseline under concomitant standard triple immunosuppressive therapy post kidney transplantation, including steroids, mycophenolate acids, and calcineurin inhibitors (< 25 on a scale of 0–700). Combined analysis of cohorts A and B showed a median difference of 12.86 (CI: −68.10 to 72.06) in disease activity score from baseline to 9 to 12 months on iptacopan treatment (Supplementary Table S3). Overlaying individual time profiles of disease activity total score (cohorts A and B) are shown in Figure 8.

**Figure 9:**
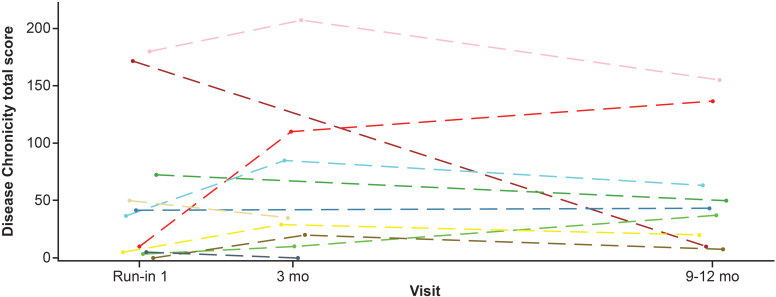
Overlaying individual time profiles (spaghetti plots) of disease chronicity biopsy total score (cohorts A and B; safety analysis set). Two patients are from cohort A; the remaining profiles are from patients in cohort B. mo, month.

A decrease in chronicity score was seen in the only patient in cohort A (this is likely to be a sampling issue) and an increase in 1 cohort B patient; whereas in the other 7, the total chronicity score remained relatively stable after 1 year (Figure 9). In the combined cohorts, iptacopan showed a comparable trend with a median difference of 6.67 (CI: −73.3 to 64.21) in disease chronicity total score from baseline to 6 to 9 months (i.e., 9‒12 months iptacopan treatment; Supplementary Table S3).

#### Biomarkers

**Figure 10:**
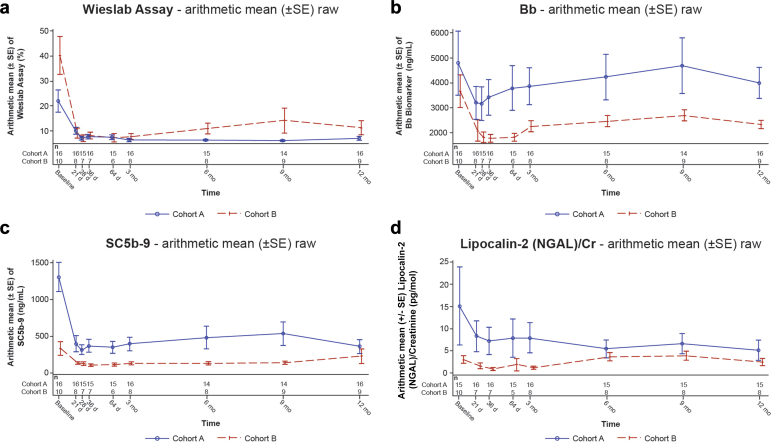
Biomarkers measured over time for cohort A and cohort B (safety analysis set)) Wieslab Assay (arithmetic mean [± SE] raw), (b) Bb (arithmetic mean [± SE] raw), (c) SC5b-9 (arithmetic mean [± SE] raw), (d) Lipocalin-2 (NGAL)/Cr (arithmetic mean [± SE] raw) for cohorts A and B. Cr, creatinine; d, day; m, month; NGAL, neutrophil gelatinase-associated lipocalin: SE, standard error; w, week.

Inhibition of the AP of the complement pathway was measured by Wieslab activity, Bb, and plasma sC5b-9 levels; tubular kidney damage was assessed by using urine lipocalin-2 (NGAL)/creatinine ratio (Figure 10). Iptacopan inhibited the AP as shown by Wieslab activity reduction (*P* < 0.0001 at 1 year of treatment in both C3G cohorts). Bb was reduced in cohort B (*P* = 0.0032) at 12 months compared to baseline. Plasma sC5b-9 levels were significantly lower for both cohorts (*P* < 0.0001 and *P* = 0.0023 in cohort A and B, respectively) at 12 months compared to baseline. Iptacopan decreased urinary NGAL/creatinine by 60% (*P* < 0.0001) in 12 months compared to baseline in cohort A.

#### Pharmacokinetics

Mean iptacopan predose plasma concentrations at 3, 6, 9, and 12 months show that iptacopan was at steady state in cohorts A and B at these study timepoints (Supplementary Table S4). Mean plasma concentrations of iptacopan were slightly higher in cohort B than in cohort A; however, pharmacokinetic concentrations from Cohort B were more variable. Iptacopan concentrations at trough in patients with C3G were similar to those in healthy volunteers.^19^

#### Safety

Most treatment-emergent adverse events (TEAEs) in each cohort were mild (Table 2). However, a greater proportion of TEAEs were moderate or severe in cohort B, possibly because of the concomitant standard triple immunosuppressive therapy post–kidney transplantation (5 and 15 TEAEs were study drug-related in cohorts A and B, respectively). One death was reported because of cardiac arrhythmia. This patient was receiving 3 agents (methylphenidate, venlafaxine, and trazadone) all of which increased risk of tachydysrhythmia; death was determined to be unrelated to iptacopan treatment. In cohort B, 3 patients had TEAEs that led to study drug interruption, and 1 patient had a serious TEAE that led to study drug discontinuation. The most common TEAEs, by preferred term, were COVID-19 (*n* = 9, 34.6%), headache (*n* = 5, 19.2%), hypertension, and vomiting (both *n* = 4, 15.4%; Supplementary Table S5).

**Table 2:** Overall summary of treatment-emergent adverse events (reported in ≥2 patients) by preferred term (safety analysis set)

Treatment-emergent adverse events	Cohort A *n* = 16 nE, nP (%)	Cohort B *n* = 10 nE, nP (%)	Overall *N* = 26 nE, nP(%)
Patients with at least one TEAE	81, 15 (93.8)	59, 9 (90.0)	140, 24 (92.3)
Severity			
Mild	74, 15 (93.8)	42, 9 (90.0)	116, 24 (92.3)
Moderate	6, 5 (31.3)	12, 5 (50.0)	18, 10 (38.5)
Severe	1, 1 (6.3)	5, 3 (30.0)	6, 4 (15.4)
Serious TEAEs	3, 2 (12.5)	6, 3 (30.0)	9, 5 (19.2)
TEAEs suspected to be related to Study drug	5, 3 (18.8)	15, 5 (50.0)	20, 8 (30.8)
Serious TEAEs suspected to be related to Study drug	0	3, 1 (10.0)	3, 1 (3.8)
TEAEs leading to study drug interruption	0	5, 3 (30.0)	5, 3 (11.5)
TEAEs leading to study drug discontinuation	0	1, 1 (10.0)	1, 1 (3.8)
Serious TEAEs leading to study drug discontinuation	0	1, 1 (10.0)	1, 1 (3.8)

*n*, number of patients studied; nE, number of TEAE events in the category; nP, number of patients with at least one TEAE in the category; TEAE, treatment-emergent adverse event.

## Discussion

We have shown that long-term exposure to iptacopan 200 mg twice daily resulted in sustained, clinically relevant proteinuria reduction from 45% at 3 months (the end of the primary phase 2 PoC study) to 57% at 12 months in patients with native C3G (cohort A), who were already receiving maximally-tolerated supportive care by means of angiotensin-converting enzyme inhibitor or angiotensin II receptor blocker therapy. At baseline, of the 9 of 16 patients with nephrotic proportion proteinuria, 6 had, after iptacopan treatment, a reduction to subnephrotic proteinuria. Nephrotic proteinuria is associated with an increased risk of thrombosis^20^ and infection,^21^ among other complications. Following completion of the phase 2 study, this extension study is important in the context of the GLOSEN group findings showing that a ≥50% proteinuria reduction during the first 6 or 12 months from diagnosis was associated with a lower risk of subsequent kidney failure.^10^

Patients with recurrent C3G (cohort B) by study design, were not required to have any albumin-/proteinuria at baseline, and urinary protein levels were largely normal (median 18.4 mg/mmol), most likely because recurrent C3G disease posttransplantation was diagnosed rather early; and possibly because of the antiproteinuric actions of the background medication such as renin-angiotensin-system blockers and calcineurin inhibitors administered to all kidney allograft recipients. Although iptacopan treatment was associated with a reduction in proteinuria in this population, this was not statistically significant.

Iptacopan treatment stabilized eGFR in both cohorts; extrapolation from historical data shows that the slope improved, suggesting a change in the clinical course of the disease. eGFR stabilization was most significant in patients with native C3G disease (cohort A), in whom eGFR deteriorated by 15.73 ml/min per 1.73 m^2^ per year over the 2 years before study start, consistent with what is known of the natural history of C3G. Once iptacopan treatment began, eGFR improved significantly, with an increase of 1.3 ml/min per 1.73 m^2^ from baseline to 12 months, equating to a predicted change of 16.6 ml/min per 1.73 m^2^ if iptacopan had not been commenced (nominal *P*-value = 0.0233). It is noteworthy to mention that if the eGFR slope had continued to be unchanged in the absence of iptacopan, the mean eGFR value would have reached 15 ml/min per 1.73 m^2^ (stage 5 chronic kidney disease) within 3.51 years. In summary, our results represent substantial preservation and even improvement of kidney function in patients with native and recurrent C3G.

Historical renal function data as measured by eGFR from the 2 years before study entry showed that patients in cohort B (eGFR slope of −5.16 ml/min per 1.73 m^2^ per year) had more stable kidney function than those in cohort A (eGFR slope of −15.73 ml/min per 1.73 m^2^ per year), reflecting the shorter duration of recurrent disease compared with native disease in patients with C3G. At 12 months’ follow-up, mean eGFR in cohort B was generally unchanged from baseline (excluding the single individual noted above). Individual patient eGFR plots show that all other patients had stable or improved kidney function over this 12-month study. A sensitivity analysis excluding the single patient who developed transplant failure showed a predicted eGFR preservation of 7.1 ml/min per 1.73 m^2^ over 1 year despite continued triple immunosuppression, including vasoconstrictive calcineurin inhibitors. These are encouraging findings given that previous studies, reviewed by Obata and colleagues have shown that C3G has a high risk of recurrence (60%–84%) after kidney transplantation.^22^

Although sample sizes were small, there was a trend toward a reduction in C3 deposition with iptacopan treatment, which is consistent with decreased alternative complement pathway activity as shown by significant reductions in the Wieslab assay and plasma sC5b-9 concentrations.

Baseline serum C3 and Wieslab activity levels were lower and sC5b-9 higher in native C3G, compared with patients with C3G post kidney transplantation and recurrent C3G disease. Serum and plasma complement biomarkers showed a pattern of substantial and persistent AP inhibition with treatment. C3 levels normalized in 8 of 16 cohort A and 7 of 9 cohort B patients, that is, achieving levels within the normal range for most patients. AP activity, measured in the Wieslab assay decreased dramatically in both cohorts as did sC5b-9 levels, most notably in cohort A.

Total activity score in kidney biopsies tended to be low at baseline in most patients in cohort B under standard triple immunosuppressive therapy. Of the 6 patients with low activity score at baseline, inflammation remained low in 5 patients after 1 year of iptacopan treatment and increased in 1 patient. Similarly, 1 patient in cohort A had a low activity score both at baseline and after 1 year of treatment. Two patients (1 from each cohort) with higher baseline activity scores had substantial decreases in their scores after 1 year of iptacopan treatment. Overall, glomerular inflammation was low and remained so for the duration of treatment.

Similarly, chronicity score in kidney biopsies tended to be low at baseline in most patients (< 50 on a scale of 0‒400) with 7 of 9 patients in both cohorts showing no, or small changes with 12 months of treatment. One patient in cohort B had an increase and 1 patient in cohort A had a decrease in chronicity score at 1 year of treatment. Overall, there was no consistent worsening of tubulo-interstitial fibrosis during the course of iptacopan treatment (median score changed from 36.7 to 43.3 over 1 year). This is relevant in the context of the knowledge that the degree of tubulo-interstitial chronicity in kidney biopsies is one of the main predictors of kidney failure in all chronic kidney disease entities, including C3G but, unfortunately considered irreversible.^8^

Urine NGAL/creatinine ratios were elevated in cohort A but not in cohort B at baseline. Notably, ratios became lower with treatment in cohort A, suggesting iptacopan can protect against kidney damage. In cohort A, the change in sC5b-9 was strongly and inversely correlated with the change in eGFR. No other biomarker correlated consistently with eGFR.

Overall, iptacopan levels both in native and recurrent C3G cohorts were similar to healthy volunteers.^19^ Iptacopan trough levels in both cohorts appear to be sufficient for sustained inhibition of the alternative complement pathway. Iptacopan concentrations were somewhat higher in cohort B compared with cohort A at all time points. No dose-adjustment of iptacopan is required for use with cyclosporine or other calcineurin inhibitors such as tacrolimus. No drug-drug interaction is expected when iptacopan is used in combination with cyclosporine or tacrolimus, prednisone or prednisolone, and mycophenolate mofetil or mycophenolate sodium.^23^ Iptacopan dose adjustment is not required in patients with mild or moderate renal impairment.^24^

Complement inhibitors are known to increase the risk of encapsulated bacterial infection as a class effect, which also applies to iptacopan. Notably, the overall safety profile of iptacopan, however, appears favorable, provided patients are vaccinated, and patients and health care professionals are diligent in monitoring for infection. Iptacopan was generally well-tolerated with most adverse events being of mild or moderate severity, similar to the safety findings of a previous study with iptacopan in paroxysmal nocturnal hemoglobinuria.^25^ There was 1 death in cohort A, as a result of cardiac arrhythmia that was not suspected to be related to iptacopan. Of note, iptacopan does not interact with other drugs^23^ and therefore there is no risk of potentiating the impact of any other drugs on QT or risk of tachyarrhythmias. One patient in cohort A had serious diarrhea and vomiting, also not suspected to be related to iptacopan. A greater proportion of adverse events in cohort B was moderate or severe, which is often seen and expected in highly immunosuppressed kidney transplant patients. Serious adverse events were reported in 3 cohort B patients, 1 of whom experienced serious pneumococcal pneumonia and sepsis requiring ventilation, suspected to be related to iptacopan, as well as calcineurin inhibitors, mycophenolate mofetil, and corticosteroid treatment; this patient recovered fully. The other 2 serious adverse events were pyelonephritis and acute kidney injury not related to iptacopan treatment.

Some limitations apply to this trial, the most important being the open-label nature of our study, the lack of a control group and the limited patient numbers. Although patient numbers in a PoC study tend to be small, this is even more so the case in an ultra-rare disease such as C3G. The limited patient numbers make it difficult to make any meaningful conclusions on the reasons behind persistently low C3 levels in some patients and any association between persistent low C3 levels and other outcomes, such as proteinuria. APPEAR-C3G, a randomized, double-blind, and placebo-controlled phase 3 study (NCT04817618) to evaluate the efficacy and safety of iptacopan in patients with C3G, is in progress. APPEAR-C3G has recruited 74 adults with biopsy-confirmed C3G with the primary objective of evaluating the efficacy of iptacopan compared with placebo on proteinuria reduction.

### Conclusion

Long-term treatment with iptacopan in native kidney C3G results in a significant and a clinically meaningful reduction in proteinuria to subnephrotic proportions, and a sustained improvement in eGFR beyond what was previously reported after 12 weeks’ treatment. Furthermore, improvement in eGFR over time was seen in recurrent C3G post kidney transplantation. Stable increases in serum C3 levels and a trend toward a reduction in C3 deposition with iptacopan treatment, consistent with decreased alternative complement pathway activity were seen in both cohorts, suggesting that treatment with iptacopan reduces overall complement activity; a circumstance that appears to stabilize kidney function, or at least delay progression toward kidney failure in patients with C3G. These results support further evaluation of iptacopan in the ongoing confirmatory phase 3 APPEAR-C3G trial (NCT04817618; recruitment complete) and strengthens the therapeutic rationale for long-term treatment of C3G with targeted inhibition of the alternative complement pathway by means of iptacopan.

## Disclosure

CMN is one of the principal investigators on this trial, has received consulting fees from Novartis, Retrophin, Biocryst, Kira, and Apellis; author royalties from UpToDate; and participated on a data safety monitoring board for Kira. UE has received payment or honoraria from Novartis, Astellas and Chiesi; and has participated in advisory boards for Novartis, Biotest, and MSD. LL is, or was recently, a speaker and/or advisor for and/or received consulting fees and/or payment for lectures from Alexion, AstraZeneca, Biogen Idec, BMS, Carna Health, GSK, Kezar, Novartis, Pfizer, and Roche; serves on a data safety monitoring board of Novartis; and has leadership roles as a Trustee of Kidney Research UK, Chair of the ISN Western EU Regional board, and is a member of the International Society of Nephrology Executive Council. MP has received payment or honoraria from Alexion, Novartis, Sanofi, Travere, Vifor, and Otsuka. GR has received consulting fees, payment or honoraria from Alexion, Novartis, Sanofi, Travere, Vifor, and Otsuka. MJS has received grants or contracts from Boehringer, Instituto, and Carlos III Marató TV3; honoraria for presenting lectures and/or support for attending meetings/travel and/or participated on a data safety monitoring committee/advisory board for NovoNordisk, Jansen, Boehringer, Mundipharma, AstraZeneca, Ingelheim Lilly, Vifor, ICU Medical, Fresenius, and Travere Therapeutics; and has leadership roles as Cardiorenal Group Spanish Society of Cardiology (SEC) group member, Big Data Spanish Society of Nephrology (SEN) Board member, ex European Renal Association (ERA) Board member, and ex editor-in-chief of Clinical Kidney Journal. EW has received payment, honoraria, or support for attending meetings and/or travel from Novartis, Alexion, and Arrowhead; and is on the data monitoring safety boards of Novartis, Alexion, Biocryst, and Apellis; he holds leadership positions as Chair of MPGN DDD and C3G Rare Disease Group of UK Kidney Association. MM and NJAW are employees of Novartis Pharma AG, Basel, Switzerland and hold stock in the company; AB and GJ are employees of Novartis Institutes for BioMedical Research, Cambridge, Massachusetts, USA and hold stock in the company; RT, AT, and JL are employees of Novartis Pharmaceuticals Corporation, East Hanover, New Jersey, USA and hold stock in the company. All the other authors declared no competing interests.

## Supplementary Material

Supplementary File (PDF) **Supplementary Methods. Supplementary Results. Figure S1.** Overlaying individual time profiles (spaghetti plots) of 24-hour UPCR by cohort (safety analysis set) (A) cohort A (B) cohort B. **Figure S2.** Model estimated geometric mean ratio to baseline (95% CI) plot of first morning void UPCR by cohort over time in cohort A (safety analysis set). **Figure S3.** Model estimated arithmetic mean change from baseline (95% CI) plot of eGFR (ml/min per 1.73 m^2^) (cohort A, safety analysis set). **Figure S4.** Overlaying individual time profiles (spaghetti plots) of C3 biomarker (g/l) (cohort A, safety analysis set). **Figure S5.** Examples of the glomerular C3 deposit scoring system based on analysis of kidney biopsy by immunofluorescence microscopy. **Table S1.** Patient disposition. **Table S2.** Subgroup analysis of patients in cohort A with “nephrotic” and “nonnephrotic” proteinuria at baseline (safety analysis set). **Table S3.** Median difference of C3 deposit score, disease activity, and chronicity total scores after 9 to 12 months of iptacopan treatment visit (Safety analysis set). **Table S4.** Predose concentrations of iptacopan in participants in cohorts A and B. **Table S5.** Treatment-emergent adverse events (reported in ≥2 participants) by preferred term. CONSORT checklist.

## Footnotes

Supplementary File (PDF)

**Supplementary Methods.**

**Supplementary Results.**
**Figure S1.** Overlaying individual time profiles (spaghetti plots) of 24-hour UPCR by cohort (safety analysis set) (A) cohort A (B) cohort B. **Figure S2.** Model estimated geometric mean ratio to baseline (95% CI) plot of first morning void UPCR by cohort over time in cohort A (safety analysis set). **Figure S3.** Model estimated arithmetic mean change from baseline (95% CI) plot of eGFR (ml/min per 1.73 m^2^) (cohort A, safety analysis set). **Figure S4.** Overlaying individual time profiles (spaghetti plots) of C3 biomarker (g/l) (cohort A, safety analysis set). **Figure S5.** Examples of the glomerular C3 deposit scoring system based on analysis of kidney biopsy by immunofluorescence microscopy. **Table S1.** Patient disposition. **Table S2.** Subgroup analysis of patients in cohort A with “nephrotic” and “nonnephrotic” proteinuria at baseline (safety analysis set). **Table S3.** Median difference of C3 deposit score, disease activity, and chronicity total scores after 9 to 12 months of iptacopan treatment visit (Safety analysis set). **Table S4.** Predose concentrations of iptacopan in participants in cohorts A and B. **Table S5.** Treatment-emergent adverse events (reported in ≥2 participants) by preferred term. CONSORT checklist.
