# Supplementary material for: Iptacopan Reduces Proteinuria and Stabilizes Kidney Function in C3 Glomerulopathy
Source: Kidney Int Rep. 2024 Oct 28;10(2):432–46. doi: 10.1016/j.ekir.2024.10.023 (PMC11843281; doi:10.1016/j.ekir.2024.10.023)
Supplement: Supplementary File (PDF) — Supplementary Methods. Supplementary Results. Figure S1. Overlaying individual time profiles (spaghetti plots) of 24-hour UPCR by cohort (safety analysis set) (A) cohort A (B) cohort B. Figure S2. Model estimated geometric mean ratio to baseline (95% CI) plot of first morning void UPCR by cohort over time in cohort A (safety analysis set). Figure S3. Model estimated arithmetic mean change from baseline (95% CI) plot of eGFR (ml/min per 1.73 m2) (cohort A, safety analysis set). Figure S4. Overlaying individual time profiles (spaghetti plots) of C3 biomarker (g/l) (cohort A, safety analysis set). Figure S5. Examples of the glomerular C3 deposit scoring system based on analysis of kidney biopsy by immunofluorescence microscopy. Table S1. Patient disposition. Table S2. Subgroup analysis of patients in cohort A with “nephrotic” and “nonnephrotic” proteinuria at baseline (safety analysis set). Table S3. Median difference of C3 deposit score, disease activity, and chronicity total scores after 9 to 12 months of iptacopan treatment visit (Safety analysis set). Table S4. Predose concentrations of iptacopan in participants in cohorts A and B. Table S5. Treatment-emergent adverse events (reported in ≥2 participants) by preferred term. CONSORT checklist. [file mmc1.pdf]

**Supplementary Methods**

*Inclusion and Exclusion Criteria*

Inclusion criteria included:

- Written informed consent was obtained before any assessment was performed
- Male and female participants 18 years of age or greater at screening
- Participants completed the treatment period of the Phase 2 proof-of-concept (PoC) trial on study drug
- Able to communicate well with the Investigator, understand and comply with the requirements of the study [applicable in Germany only]
- Participants or their legal representatives who were able to communicate well with the Investigator, understand and comply with the requirements of the study [applicable in all countries except Germany]

Key exclusion criteria included

- Severe concurrent co-morbidities, e.g., advanced cardiac disease [New York Heart Association (NYHA) class IV], severe pulmonary arterial hypertension [World health Organization (WHO) class IV], or any illness or medical condition that in the opinion of the Investigator and sponsor is likely to prevent the participants from safely tolerating iptacopan or complying with the requirements of the study
- Participants with an active systemic bacterial, viral, or fungal infection within 14 days prior to screening, or the presence of fever ≥ 38$^{o}$C (100.4$^{o}$F) within 7 days prior to screening
- History of human immunodeficiency virus (HIV) or any other immunodeficiency disease
- History or current diagnosis of electrocardiogram (ECG) abnormalities indicating significant risk of safety for participants

*Histopathology*

Histologic Scores for Disease Activity and Disease Chronicity Scores

The histologic activity and chronicity scores integrate the parameters described by Bomback et al.[8], by grading as a percentage range (number of glomeruli affected divided by the number of total glomeruli present in the slides). The scores designed in this way allowed higher resolution as compared to the 0–3 graded scale from the reference. The scores were based on standard light microscopy staining (periodic acid-Schiff, hematoxylin and eosin, and Jones' stain).

The activity score was based on the following 7 parameters, hence ranging from 0–700:

- Mesangial hypercellularity
- Endocapillary hypercellularity
- Glomerular basement membrane double contours
- Cellular or fibrocellular crescent
- Necrosis
- Interstitial inflammation in non-scarred cortex
- Leukocytes in the glomeruli

The chronicity score was based on the following 4 parameters, hence ranging from 0–400:

- Segmental or global sclerosis
- Fibrous crescents
- Tubulointerstitial fibrosis
- Significant vascular sclerosis present (score of >50% intima vs media thickness)

Definitions were as per Bomback et al.[8] and Bajema et al.[17]

- Mesangial hypercellularity is defined as >3 nuclei in the mesangium surrounded by matrix away from the hilum
- Endocapillary hypercellularity is defined by increased neutrophils, or combined neutrophils and/or macrophages in capillary lumens. It is scored when the capillary lumen is expanded by the cellular process

- Arteriosclerosis is defined as 0 if vascular lesions were absent or as 1 if intimal thickening > thickness of media

**Statistical hypothesis, model, and method of analysis**

*Analysis of primary endpoints*

1. Cohort A: The statistical evaluation of all primary efficacy data was descriptive. Hypothesis testing was not performed.
2. Cohort B: The Wilcoxon signed rank test was used for C3 Deposit Score data at the 6- to 9-month visit in the extension study to compare the median difference of change from baseline between periods. The Hodges-Lehmann estimate and two-sided 95% confidence interval (CI) for the median difference were provided

*Analysis of secondary endpoints*

The safety (SAF) analysis set was included for the secondary analyses

1. Cohort A: The number of participants meeting the requirements of the composite kidney endpoint 2 at the Day 7, Day 14, Day 21, Day 28, Day 36, Day 64 and Day 84 visits in the Phase 2 PoC study and at the 3-month, 6-month, 9 month and 12-month visits of extension study (with the 9-month visit being of primary interest)
2. Raw and ratio to baseline in first morning void (FMV) urine protein-creatinine ratio (UPCR) at the Day 7, Day 14, Day 21, Day 36 and Day 64 visits in the core study and at the 3-month, 6-month, 9 month and 12-month visits in the extension study
3. Raw and ratio to baseline in 24-hour UPCR at the Day 28 and Day 84 visits in the Phase 2 PoC study and at the 9 month visit in the extension study
4. Raw and change from baseline in eGFR at the Day 7, Day 14, Day 21, Day 28, Day 36, Day 64 and Day 84 visits in the Phase 2 PoC study and at the 3-month, 6-month, 9 month and 12-month visits in extension study
5. Cohort B: Change from baseline in the disease activity and chronicity scores (based on light microscopy) compared to baseline in the study at the Day 84 visit in the Phase 2 PoC study and at the 6- to 9-month visit in extension study

*Analysis of end point exploring the historical eGFR data in Cohort A*

A generalized linear mixed model, with a common intercept, a pre-treatment slope, a change in the slope following iptacopan treatment, and cohort were used to predict the pre-post iptacopan change in eGFR over time. eGFR slope prior to iptacopan treatment and the change in eGFR slope after iptacopan treatment was explored and presented graphically.

*Activity Score*

The activity score has 7 individual scoring domains (mesangial hypercellularity, endocapillary hypercellularity, glomerular basement membrane double contours, cellular or fibrocellular crescents, necrosis, interstitial inflammation in non-scarred cortex, leukocytes in the glomeruli).

*Chronicity Score*

The chronicity score has 4 individual scoring domains (segmental or global sclerosis, fibrous crescents, percent interstitial fibrosis/tubular atrophy and arteriosclerosis present [score of 100% intima vs media thickness]).

*Modelling*

Log-transformed ratio to baseline in 24-hour UPCR values at the three post-baseline time points of were analyzed separately using a mixed model repeated measures (MMRM) model with time point (as study day relative to the date of first administration of study treatment) as a fixed effect, and the Phase 2 (PoC) log-transformed baseline measurement as fixed covariate. Log-transformed ratio to baseline in FMV UPCR and values at the nine post-baseline time points of interest were analyzed separately using a MMRM model with time point (as study day relative to the date of first administration of study treatment) as a fixed effect, and the Phase 2 (PoC) log-transformed baseline measurement as fixed covariate. Change from baseline in eGFR values at eleven post-baseline time points of interest were analyzed separately using a MMRM model with time point (as study day relative to the date of first administration of study treatment) as a fixed effect, and the Phase 2 (PoC) baseline measurement as fixed covariate.

MMRM models were fitted using all available data including data collected after treatment discontinuation. This method is valid and consistent under a ‘missing at random’ (MAR) assumption, i.e., given the

observed data (responses and covariates) the probability of drop-out does not depend on the unobserved responses.

## Supplementary Results

*Patient disposition*

Twenty-six participants from the Phase 2 (PoC) study (Day 84 for 25 participants and Day 168 for one participant), willing to continue taking iptacopan entered into the extension study (none were screen failures) according to their previous cohorts (A/B).

Twenty-five participants completed the 12-month visit (9 months in extension), and 22 participants were ongoing in the extension study at the time of this report. Four participants discontinued. The reasons for discontinuation from treatment were death, adverse event (AE), physician decision and subject decision in one participant each.

*Change from baseline in eGFR*

eGFR data were collected from study patients over the 2 years before their entry into the study or from diagnosis where this was less than 2 years. This showed that the mean pre-study eGFR slope was -15.73 ml/min/1.73m$^2$/year (p=0.0014), consistent with the known natural history of native C3G. Following treatment with iptacopan for one year an improvement in eGFR of 1.3 ml/min/1.73m$^2$ from baseline was observed (Note that the eGFR improvement value of 1.3 ml/min/1.73m$^2$, which was considered and affected by all pre- and post-treatment datapoints in the analysis, was less than the improvement in eGFR (6.83 ml/min/1.73m$^2$) by considering only the baseline and post-treatment datapoints). This equated to the predicted preservation of 16.6 ml/min/1.73m$^2$ (p=0.0233) of eGFR over one year compared with the scenario where iptacopan had not been commenced and the eGFR continued to deteriorate at the same rate.

## Supplementary Figures

**Supplementary Figure S1** Overlaying individual time profiles (spaghetti plots) of 24-h UPCR by cohort (safety analysis set) (a) Cohort A (b) Cohort B

(a)

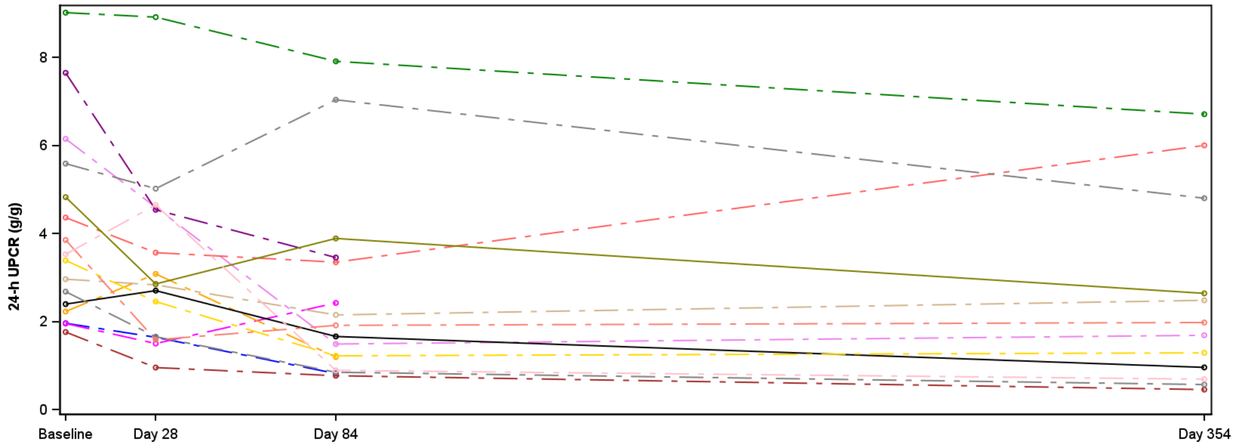


**(b)**

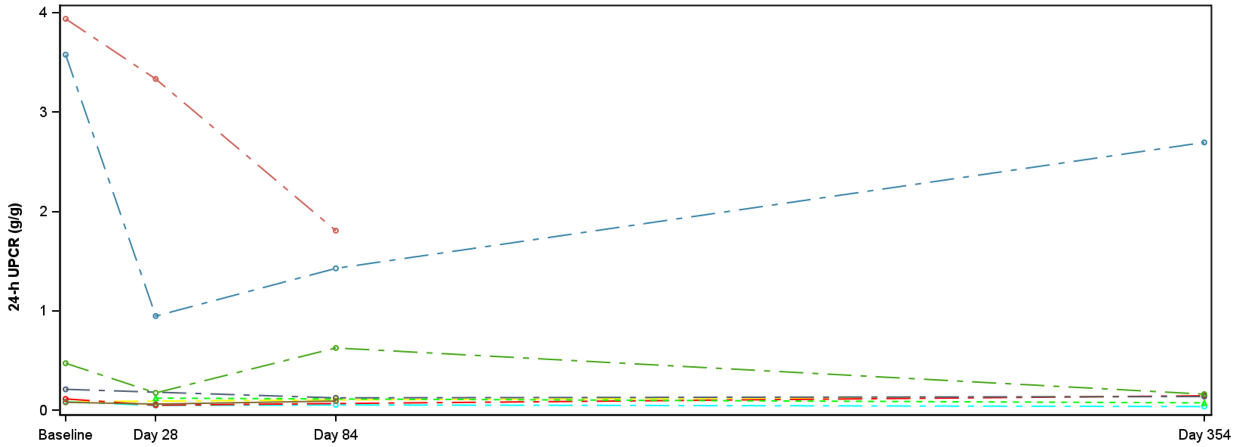

**Supplementary Figure S2** Model estimated geometric mean ratio to baseline (95% CI) plot of FMV UPCR by cohort over time in Cohort A (safety analysis set).

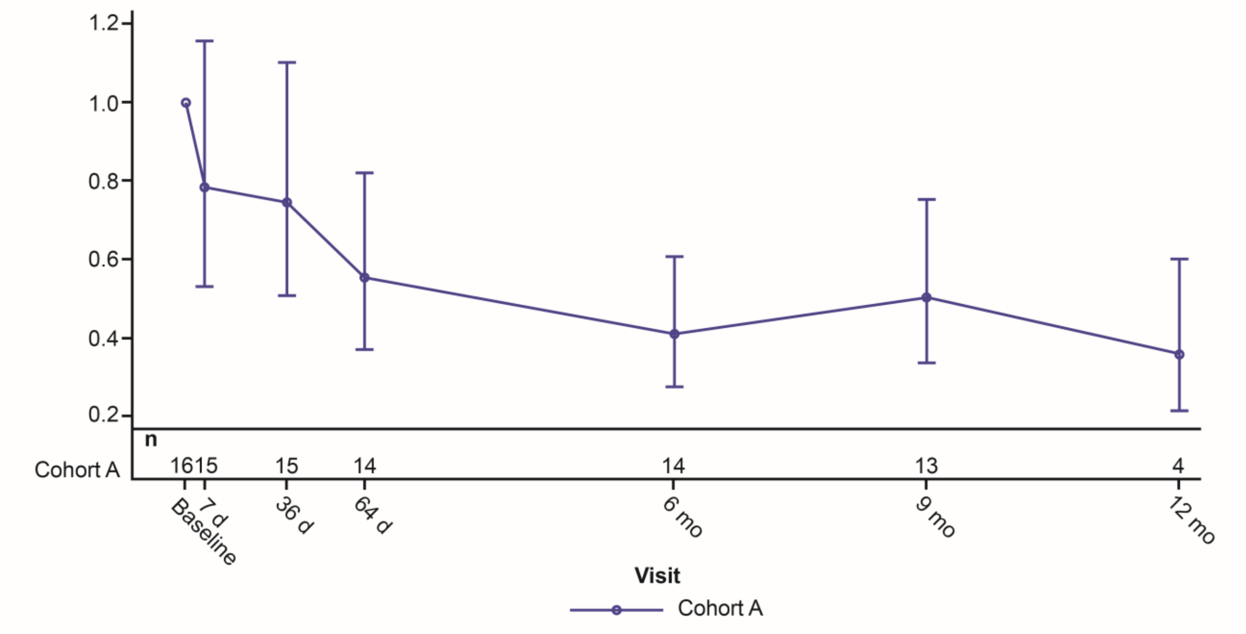


CI, confidence interval; d, day; FMV, first morning void; mo, month; UPCR, urine protein–creatinine ratio.

**Supplementary Figure S3** Model estimated arithmetic mean change from baseline (95% CI) plot of eGFR (mL/min/1.73 $m^2$) (Cohort A, safety analysis set).

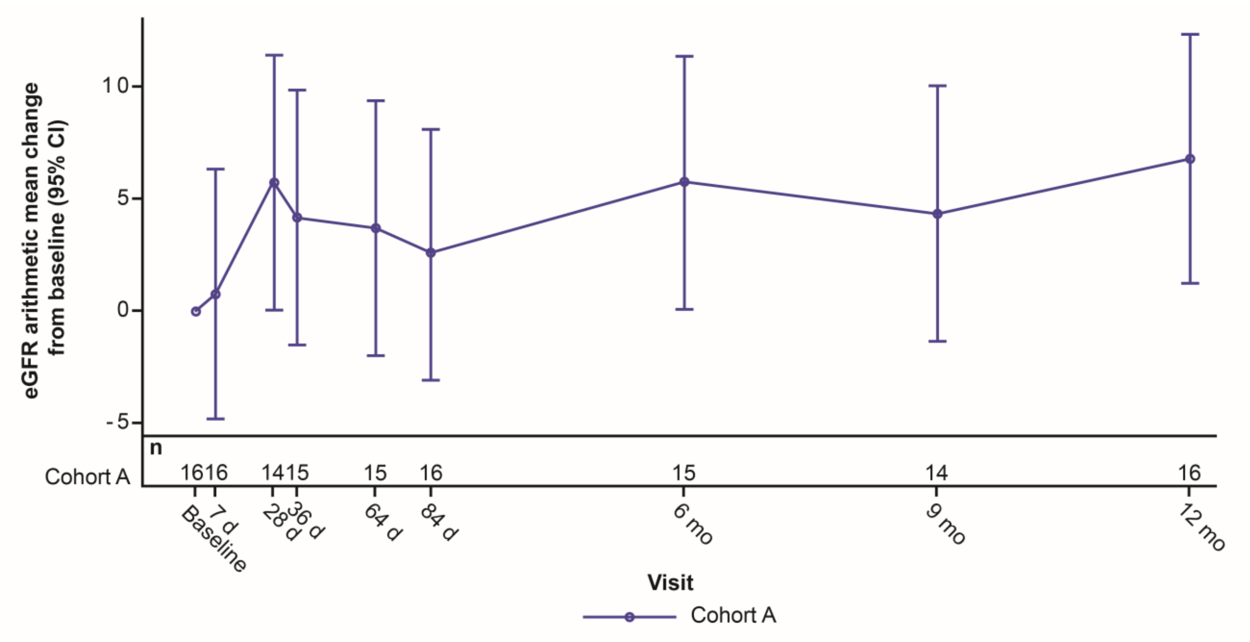


CI: confidence interval; d: day; eGFR: glomerular filtration rate; mo: month

**Supplementary Figure S4** Overlaying individual time profiles (spaghetti plots) of C3 biomarker (g/L) (Cohort A, safety analysis set).

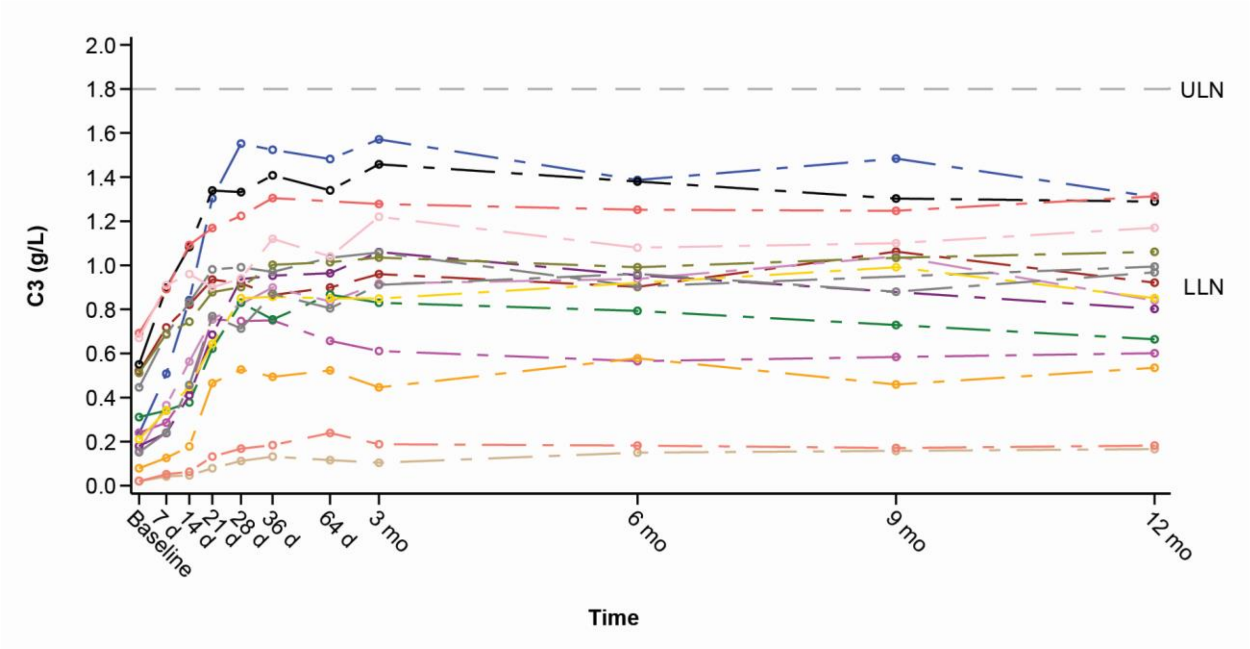


d, day; mo, month; LLN, lower limit of normal; ULN, upper limit of normal.

**Supplementary Figure S5** Examples of the glomerular C3 deposit scoring system based on analysis of kidney biopsy by immunofluorescence microscopy**.** (a) shows segmental 2+ mesangial and capillary granular staining; (b) shows 3+ global granular capillary and mesangial staining; (c) shows 2+ global mesangial and 1+ segmental (less than 50% of loops) granular capillary staining; (d shows 3+ global mesangial and  1+ very segmental capillary granular staining.

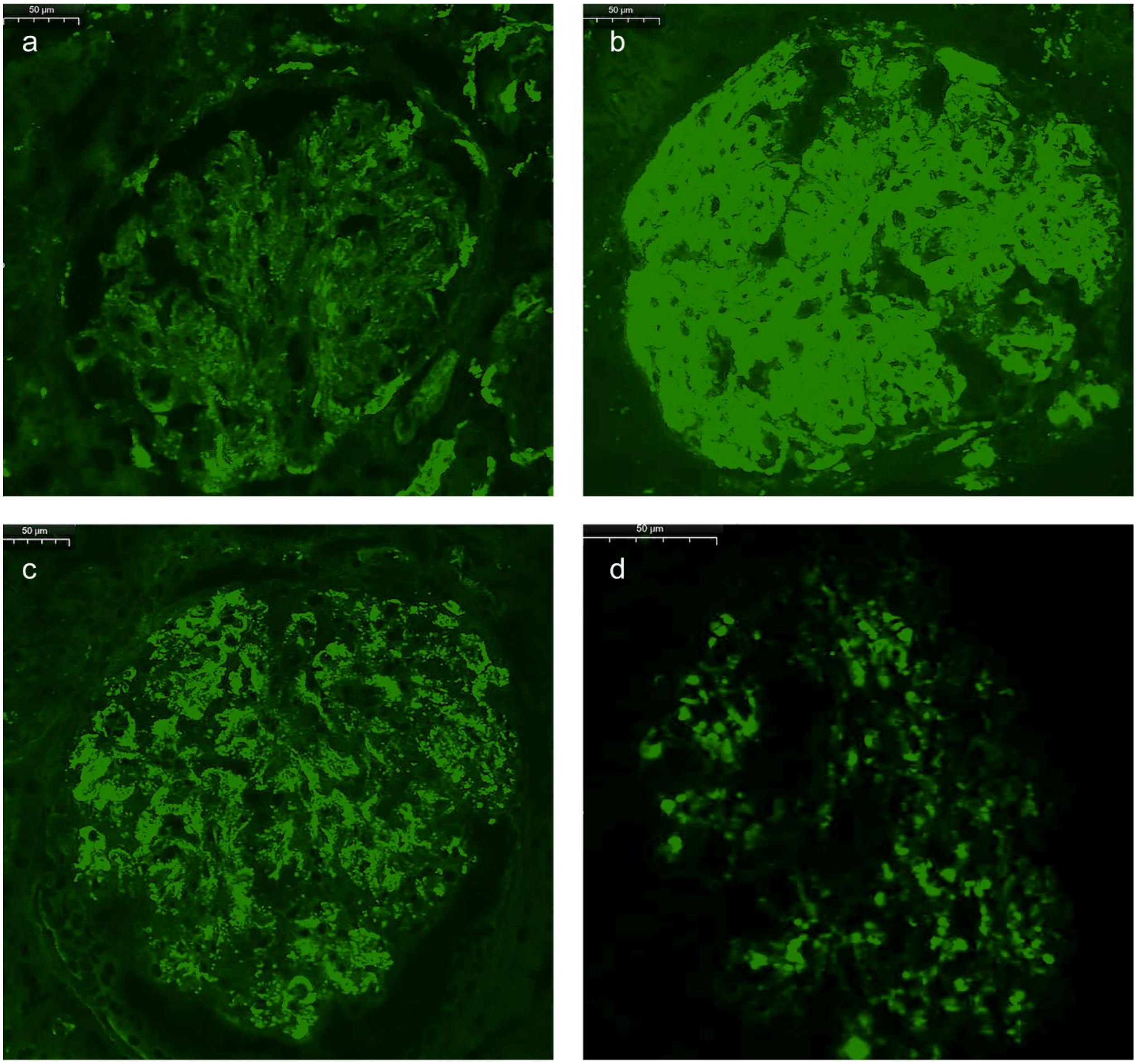

## Supplementary Tables

**Supplementary Table S1** Patient disposition.

| | Cohort A N=16 n (%) | Cohort B N=10 n (%) | Overall N=26 n (%) |
|---|---|---|---|
| **Participants** | | | |
| Enrolled (Entered the extension study and not screen failed) | 16 (100.0) | 10 (100.0) | 26 (100.0) |
| Completed 12-month visit (9 month in extension) | 16 (100.0) | 9 (90.0) | 25 (96.2) |
| Completed 15-month visit (12 month in extension) | 15 (93.8) | 8 (80.0) | 23 (88.5) |
| Ongoing (active in the study) | 14 (87.5) | 8 (80.0) | 22 (84.6) |
| Discontinued | 2 (12.5) | 2 (20.0) | 4 (15.4) |
| **Main cause of discontinuation** | | | |
| Adverse Event | 0 (0.0) | 1 (10.0) | 1 (3.8) |
| Death* | 1 (6.3) | 0 (0.0) | 1 (3.8) |
| Physician Decision | 0 (0.0) | 1 (10.0) | 1 (3.8) |
| Subject Decision | 1 (6.3) | 0 (0.0) | 1 (3.8) |

N, number of participants in the screened analysis set.

n, number of participants under each disposition category.

* There was one death in Cohort A, as a result of cardiac arrhythmia that was not suspected to be related to iptacopan.

**Supplementary Table S2** Subgroup analysis of patients in Cohort A with 'nephrotic' and 'non-nephrotic' proteinuria at baseline (safety analysis set).

| Cohort | Subgroup | Timepoint | N | n | Unadjusted geometric mean ratio to baseline (CV%) | Adjusted geometric mean ratio to baseline (95% CI)* | p-value* |
|---|---|---|---|---|---|---|---|
| Cohort A | Baseline UPCR 24hr ≤ 339 g/mol | Day 28 | 7 | 7 | 0.85 (33.7) | 0.85 (0.51, 1.43) | 0.5187 |
| | | Day 84 | 7 | 7 | 0.57 (47.4) | 0.57 (0.34, 0.96) | 0.0357 |
| | | **Day 354** | **7** | **6** | **0.38 (125.1)** | **0.38 (0.22, 0.68)** | **0.0026** |
| | Baseline UPCR 24hr > 339 g/mol | Day 28 | 9 | 8 | 0.75 (37.2) | 0.72 (0.46, 1.12) | 0.1359 |
| | | Day 84 | 9 | 9 | 0.53 (62.7) | 0.53 (0.35, 0.82) | 0.0067 |
| | | **Day 354** | **9** | **9** | **0.45 (83.8)** | **0.45 (0.29, 0.69)** | **0.0012** |

MMRM model analysis of log-transformed ratio to baseline in 24-h UPCR by baseline proteinuria subgroup in Cohort A. Heavy proteinuria at baseline was defined as 24-h UPCR >339 g/mol (3 g/g); less heavy proteinuria was defined as 24-h UPCR ≤339 g/mol.

The 9-month visit (Day 354 of treatment) is the primary interest visit and marked in bold. Log transformed ratio to baseline is analyzed using a MMRM model. Unadjusted Geo-mean is calculated based on the raw data; adjusted Geo-mean is calculated based on MMRM model. 24-h UPCR values were imputed by their FMV values at Day 354 for participants with missing 24-h UPCR values at Day 354.

Baseline is defined as the 24-h urine collection on Day -1 to Day 1. N, number of participants in treatment group. n, number of participants in each treatment group at each visit. *Calculated from two-sided test.

**Supplementary Table S3**

Median difference of C3 deposit score, disease activity and chronicity total scores after 9–12 months of iptacopan treatment visit (Safety analysis set).

| | Medians | | | Difference | | |
|---|---|---|---|---|---|---|
| **Cohort** | **n** | **Median Baseline** | **Median 6-9 months in CLNP023B12001B** | **Median difference (90% CI)** | **Shift Location** | **p-value** |
| C3 deposit score | | | | | | |
| Cohort B only | 4 | 7.50 | 2.00 | -5.50 (-10.0, 4.00) | -3 | 0.3750 |
| Cohort A+B | 5 | 9.00 | 2.00 | -7.00 (-12.0, 4.00) | -5.5 | 0.1875 |
| Disease activity total score | | | | | | |
| Cohort A+B | 9 | 7.14 | 20.00 | 12.86 (-68.1, 72.06) | 0.5 | 1.0000 |
| Disease chronicity total score | | | | | | |
| Cohort A+B | 9 | 36.67 | 43.33 | 6.67 (-73.3, 64.21) | 4.5 | 0.6523 |

The Wilcoxon signed rank test was used for C3 Deposit Score, disease activity total score data disease chronicity total score data at the 6–9 month visit in the extension study to compare the median difference of change from baseline between periods.

n: number of participants with non-missing change from baseline measurements.

**Supplementary Table S4** Iptacopan pre-dose concentrations in participants in Cohorts A and B.

| | Day 84 (3 months) | | Day 174 (6 months) | | Day 264 (9 months) | | Day 354 (12 months) | |
|---|---|---|---|---|---|---|---|---|
| | Cohort A | Cohort B | Cohort A | Cohort B | Cohort A | Cohort B | Cohort A | Cohort B |
| n | 16 | 8 | 15 | 8 | 13 | 7 | 13 | 8 |
| Mean concentration, ng/mL (SD) | 1640 (802) | 2030 (1050) | 1600 (722) | 2480 (1830) | 1760 (805) | 2850 (1650) | 1600 (594) | 2520 (1480) |
| CV% mean | 49.0 | 51.7 | 45.2 | 73.9 | 45.7 | 58.0 | 37.1 | 58.8 |

CV%, coefficient of variation (%) = (sd/mean) *100; SD, standard deviation.

**Supplementary Table S5** Treatment-emergent AEs (reported in ≥2 participants) by preferred term.

| Preferred term | Cohort A N=16 n (%) | Cohort B N=10 n (%) | Overall N=26 n (%) |
|---|---|---|---|
| Participants with at least one TEAE | 15 (93.8) | 9 (90.0) | 24 (92.3) |
| **Preferred term** | | | |
| COVID-19 | 4 (25.0) | 5 (50.0) | 9 (34.6) |
| Headache | 2 (12.5) | 3 (30.0) | 5 (19.2) |
| Hypertension | 2 (12.5) | 2 (20.0) | 4 (15.4) |
| Vomiting | 2 (12.5) | 2 (20.0) | 4 (15.4) |
| Blood Creatine Phosphokinase Increased | 2 (12.5) | 1 (10.0) | 3 (11.5) |
| Nasopharyngitis | 2 (12.5) | 1 (10.0) | 3 (11.5) |
| Proteinuria | 2 (12.5) | 1 (10.0) | 3 (11.5) |
| Abdominal Pain Upper | 2 (12.5) | 0 | 2 (7.7) |
| Anaemia | 1 (6.3) | 1 (10.0) | 2 (7.7) |
| Hyperkalaemia | 1 (6.3) | 1 (10.0) | 2 (7.7) |
| Influenza | 1 (6.3) | 1 (10.0) | 2 (7.7) |
| Iron Deficiency | 2 (12.5) | 0 | 2 (7.7) |
| Lipase Increased | 1 (6.3) | 1 (10.0) | 2 (7.7) |
| Metabolic Acidosis | 1 (6.3) | 1 (10.0) | 2 (7.7) |
| Pain in Extremity | 2 (12.5) | 0 | 2 (7.7) |
| Renal Impairment | 2 (12.5) | 0 | 2 (7.7) |
| Rhinorrhoea | 1 (6.3) | 1 (10.0) | 2 (7.7) |
| Urinary Tract Infection | 1 (6.3) | 1 (10.0) | 2 (7.7) |

A participant with multiple TEAEs is counted only once in the “at least one TEAE” row and a participant with multiple TEAEs with the same preferred term is counted only once for that preferred term and cohort. Table arranged in descending order of frequency (in overall group) and alphabetically by preferred term.

COVID-19, coronavirus disease of 2019; TEAE, treatment-emergent adverse event.

**CONSORT Checklist**

| Section/Topic | Item No | Checklist item | Reported on page no. |
|---|---|---|---|
| **Title and abstract** | | | |
| | 1a | Identification as a randomised trial in the title | n/a |
| | 1b | Structured summary of trial design, methods, results, and conclusions (for specific guidance see CONSORT for abstracts) | n/a (extension study of previously published study)[16] |
| **Introduction** | | | |
| Background and objectives | 2a | Scientific background and explanation of rationale | 4/5 |
| | 2b | Specific objectives or hypotheses | 5 |
| **Methods** | | | |
| Trial design | 3a | Description of trial design (such as parallel, factorial) including allocation ratio | 6 |
| | 3b | Important changes to methods after trial commencement (such as eligibility criteria), with reasons | n/a |
| Participants | 4a | Eligibility criteria for participants | 6 |
| | 4b | Settings and locations where the data were collected | n/a |
| Interventions | 5 | The interventions for each group with sufficient details to allow replication, including how and when they were actually administered | 5/previous publication[16] |
| Outcomes | 6a | Completely defined pre-specified primary and secondary outcome measures, including how and when they were assessed | 5/6/ previous publication[16] |
| | 6b | Any changes to trial outcomes after the trial commenced, with reasons | n/a |
| Sample size | 7a | How sample size was determined | Previous publication[16] |
| | 7b | When applicable, explanation of any interim analyses and stopping guidelines | 8/Figure 1 |
| Randomisation: | | | |
| Sequence generation | 8a | Method used to generate the random allocation sequence | n/a |
| | 8b | Type of randomisation; details of any restriction (such as blocking and block size) | n/a |

| | | | |
|---|---|---|---|
| Allocation concealment mechanism | 9 | Mechanism used to implement the random allocation sequence (such as sequentially numbered containers), describing any steps taken to conceal the sequence until interventions were assigned | n/a |
| Implementation | 10 | Who generated the random allocation sequence, who enrolled participants, and who assigned participants to interventions | n/a |
| Blinding | 11a | If done, who was blinded after assignment to interventions (for example, participants, care providers, those assessing outcomes) and how | n/a |
| | 11b | If relevant, description of the similarity of interventions | n/a |
| Statistical methods | 12a | Statistical methods used to compare groups for primary and secondary outcomes | 8/9 |
| | 12b | Methods for additional analyses, such as subgroup analyses and adjusted analyses | 6 |
| **Results** | | | |
| Participant flow (a diagram is strongly recommended) | 13a | For each group, the numbers of participants who were randomly assigned, received intended treatment, and were analysed for the primary outcome | n/a |
| | 13b | For each group, losses and exclusions after randomisation, together with reasons | n/a |
| Recruitment | 14a | Dates defining the periods of recruitment and follow-up | |
| | 14b | Why the trial ended or was stopped | n/a |
| Baseline data | 15 | A table showing baseline demographic and clinical characteristics for each group | 27/28 |
| Numbers analysed | 16 | For each group, number of participants (denominator) included in each analysis and whether the analysis was by original assigned groups | 10/ previous publication[16] |
| Outcomes and estimation | 17a | For each primary and secondary outcome, results for each group, and the estimated effect size and its precision (such as 95% confidence interval) | 10-12 |
| | 17b | For binary outcomes, presentation of both absolute and relative effect sizes is recommended | n/a |
| Ancillary analyses | 18 | Results of any other analyses performed, including subgroup analyses and adjusted analyses, distinguishing pre-specified from exploratory | 11 |

| | | | |
|---|---|---|---|
| Harms | 19 | All important harms or unintended effects in each group (for specific guidance see CONSORT for harms) | 14 |
| **Discussion** | | | |
| Limitations | 20 | Trial limitations, addressing sources of potential bias, imprecision, and, if relevant, multiplicity of analyses | 18 |
| Generalisability | 21 | Generalisability (external validity, applicability) of the trial findings | 18 |
| Interpretation | 22 | Interpretation consistent with results, balancing benefits and harms, and considering other relevant evidence | 18 |
| **Other information** | | | |
| Registration | 23 | Registration number and name of trial registry | clinical trials.gov NCT03955441, 6 |
| Protocol | 24 | Where the full trial protocol can be accessed, if available | n/a |
| Funding | 25 | Sources of funding and other support (such as supply of drugs), role of funders | 20 |
